# Foxm1 regulates neural progenitor fate during spinal cord regeneration

**DOI:** 10.15252/embr.202050932

**Published:** 2021-08-24

**Authors:** Diane Pelzer, Lauren S Phipps, Raphael Thuret, Carlos J Gallardo‐Dodd, Syed Murtuza Baker, Karel Dorey

**Affiliations:** ^1^ Division of Developmental Biology and Medicine Faculty of Biology, Medicine and Health School of Medical Sciences University of Manchester Manchester UK; ^2^ Division of Informatics Imaging & Data Sciences Lydia Becker Institute of Immunology and Inflammation Faculty of Biology, Medicine and Health School of Biological Sciences University of Manchester Manchester UK; ^3^ Present address: Institut Curie Sorbonne Université, CNRS UMR3215, INSERM U934 PSL Research University Paris France; ^4^ Present address: Department of Microbiology, Tumor and Cell Biology Science for Life Laboratory Karolinska Institutet Solna Sweden

**Keywords:** differentiation, Foxm1, regeneration, spinal cord, *Xenopus*, Development & Differentiation, Neuroscience, Regenerative Medicine

## Abstract

*Xenopus* tadpoles have the ability to regenerate their tails upon amputation. Although some of the molecular and cellular mechanisms that globally regulate tail regeneration have been characterised, tissue‐specific response to injury remains poorly understood. Using a combination of bulk and single‐cell RNA sequencing on isolated spinal cords before and after amputation, we identify a number of genes specifically expressed in the spinal cord during regeneration. We show that Foxm1, a transcription factor known to promote proliferation, is essential for spinal cord regeneration. Surprisingly, Foxm1 does not control the cell cycle length of neural progenitors but regulates their fate after division. In *foxm1* ^−/−^ tadpoles, we observe a reduction in the number of neurons in the regenerating spinal cord, suggesting that neuronal differentiation is necessary for the regenerative process. Altogether, our data uncover a spinal cord‐specific response to injury and reveal a new role for neuronal differentiation during regeneration.

## Graphical abstract

Differentiation of neural progenitor cells (NPCs) is a hallmark of successful spinal cord regeneration. This study shows that Foxm1 controls the switch from proliferation to differentiation of NPCs during spinal cord regeneration in *Xenopus tropicalis*.

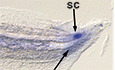

## Introduction

Mammals have limited tissue regeneration capabilities, particularly in the case of the central nervous system. Spinal cord injuries (SCIs) are often irreversible and lead to the loss of motor and sensory function below the site of the damage (McDonald & Sadowsky, 2002). In contrast, amphibians such as *Xenopus* (*X.*) tadpoles have far greater regenerative abilities as they can regenerate a fully functional tail following amputation, including their spinal cord (Deuchar, 1975; Love *et al*, 2011; Kakebeen *et al*, 2020). The injured spinal cord is sealed within a day by the formation of the neural ampulla, and lineage tracing has revealed that the spinal cord regenerates from its original stump (Gargioli & Slack, 2004; Slack *et al*, 2008). A hallmark of spinal cord regeneration is the re‐activation of Sox2/3^+^ neural progenitor cells (NPCs) to induce both regrowth of the spinal cord and the generation of new neurons (Muñoz *et al*, 2015). In axolotl, the spinal cord regrows from extensive proliferation of NPCs located in the “source zone” adjacent to the site of amputation (Mchedlishvili *et al*, 2007; Rost *et al*, 2016). This increase in proliferation is tightly regulated as progenitors switch from a neurogenic to a proliferative division. A key factor driving that switch is the planar cell polarity (PCP) pathway which is re‐activated in the spinal cord following amputation (Rodrigo Albors *et al*, 2015). However, how the balance between self‐renewal proliferation and differentiation is controlled during regeneration is currently not well understood.

During development, the switch from a proliferative to a neurogenic division depends at least in part on changes in the relative length of the different phases of the cell cycle (Cheffer *et al*, 2013; Hardwick & Philpott, 2014). We therefore took an unbiased approach to identify cell cycle regulators expressed specifically during *X*. *tropicalis* spinal cord regeneration by RNA‐seq. This led to the identification of Foxm1, a transcription factor known to promote G2/M transition, as a potential key transcription factor for spinal cord regeneration. *Foxm1* ^−/−^ *X*. *tropicalis* tadpoles develop normally but their ability to regenerate their spinal cords is impaired. Using single‐cell (sc)RNA‐seq and immunolabelling experiments, we show that *foxm1* ^+^ cells in the regenerating spinal cord undergo a transient but dramatic change in the relative proportions of cells in different phases of the cell cycle, suggesting a change in their ability to differentiate. Surprisingly, Foxm1 does not regulate the rate of progenitor proliferation or the length of the cell cycle but is required for neuronal differentiation leading to successful spinal cord regeneration.

## Results

### Foxm1 is specifically expressed in the regenerating spinal cord

**Figure 1:**
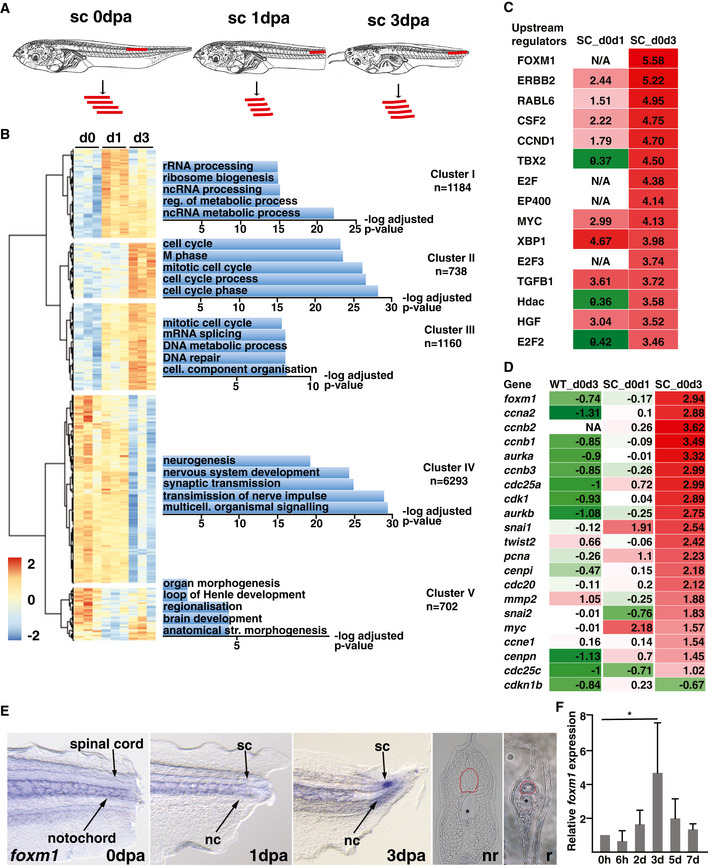
Analysis of differentially expressed genes during spinal cord regeneration ATwenty spinal cords of NF50 tadpoles were isolated at 0, 1 and 3 days post‐amputation (dpa) and pooled for RNA sequencing.BGenes with a |log2(FC)|> 1 and *P*‐adj< 0.01 were used for hierarchical clustering. For each cluster, the gene list was uploaded on Fidea (http://circe.med.uniroma1.it/fidea/) (D’Andrea *et al*, 2013). The five most significant enrichment of GO (biological processes) terms are shown, and the −log10(*P*_value) with Bonferroni correction is shown.CThe dataset was uploaded on the Ingenuity Pathway Analysis software (Qiagen). Genes with a |log2(FC)|> 1 and *P*_adj < 0.01 were considered. The software identified upstream regulators based on the changes in expression levels of known downstream targets. Each upstream regulator is attributed a z‐score, corresponding to the negative log of the *P*‐value derived from the Fisher’s exact test.DChanges in the expression of *foxm1* and known downstream targets in the whole tail comparing day 0 and day 3 (WT_d0d3) and in the spinal cord comparing day 0 and day 1 (SC_d0d1) and day 0 and day 3 (SC_d0d3). The whole tail dataset was obtained from (Chang *et al*, 2017).ETadpoles at NF50 were amputated, fixed at the indicated time and then processed for whole‐mount in situ hybridisation using a probe specific for *foxm1*. The two last panels show transverse section in the non‐regenerating spinal cord (nr) and the regenerate (r) at 3dpa. The red circle highlights the spinal cord and the asterisk the notochord.FTotal RNA was isolated from regenerating tails at indicated timepoints post‐amputation, reverse‐transcribed into cDNA and analysed for *foxm1* expression by qPCR, using *ef1*α as a reference gene. The graph represents the mean ± SD of three independent experiments. One‐way ANOVA with Dunnett’s multiple comparison test was used. *P = 0.0149.

**Figure EV1:**
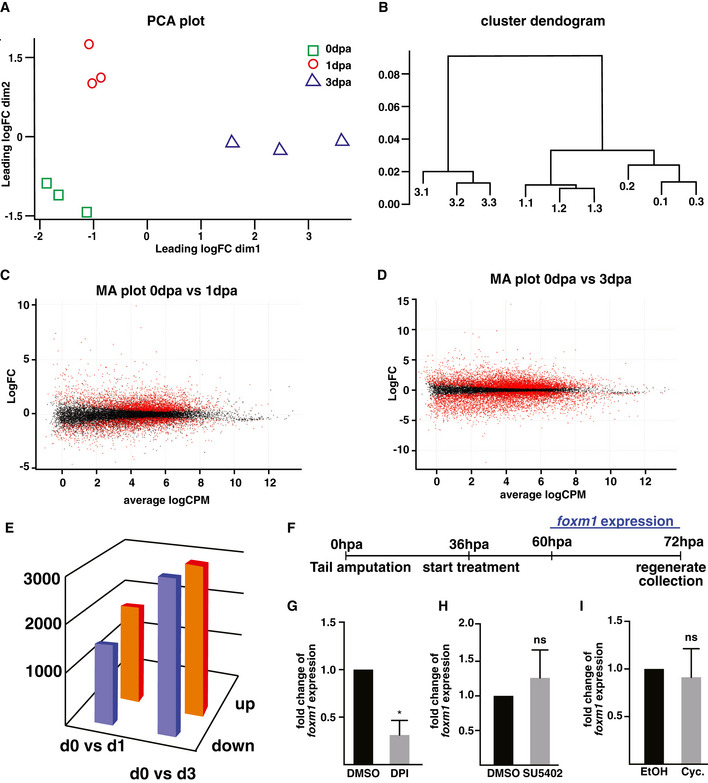
Metadata from the RNA‐seq experiment of the time course of isolated spinal cord regeneration APrincipal component analysis to assess overall similarities between all samples. The biological replicates of day 0 (0 dpa, green square), day 1 (1 dpa, red circle) and day 3 post‐amputation (3 dpa, blue triangle) cluster together whilst showing wide variation in the two dimensions shown on the graph.BHierarchical clustering of the nine datasets.C, DMA plots depicting the log2 fold change against the mean of normalised counts. DE genes (*P*_adj< 0.05) are coloured in red when comparing day 0 versus day 1 (C) and day 0 versus day 3 (D).ETotal number of differentially up‐ and downregulated (|Log2(FC)|> 1, *P*_adj< 0.01) transcripts in 0 dpa versus 1 dpa and 0 dpa versus 3 dpa samples.FSchematic of the experiment designed to identify the signals upstream of *foxm1* expression. After amputation, the tails were left to heal for 36h before inhibitor treatments were started. The tails were collected at 72hpa, and *foxm1* expression was determined by RT–qPCR.G–IEffects of treating tadpoles with 4 µM DPI (a NOX inhibitor, G), 20 µM SU5402 (an FGFR inhibitor, H) and 2.5 µM cyclopamine (a Hedgehog signalling inhibitor, I) on *foxm1* expression. DMSO was used as a control for G and H and ethanol for I. Data presentation: The graphs in G–I represent the mean with standard deviation of four independent experiments with at least 15 tails per experiments, *ef1*α was used to normalise expression, and significance was assessed with an unpaired *t*‐test, *P < 0.05.

**Figure EV2:**
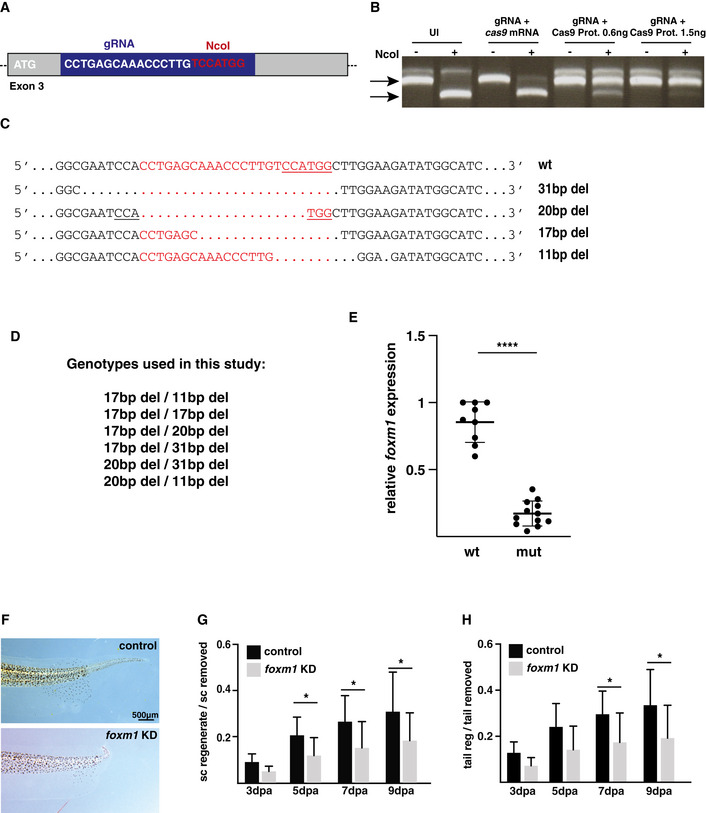
Establishment of a *foxm1* knockout line AThe CRISPR/Cas9 system was used to generate *foxm1* knockdown and knockout animals, and gRNA was designed to target the *foxm1* gene. The target region contains the restriction site for NcoI and was used to test efficiency by RFLP.BEmbryos were either uninjected (UI) or coinjected with gRNA and Cas9mRNA, 0.6 ng Cas9 protein or 1.5 ng Cas9 protein. Genomic DNA was extracted and a region amplified around the gRNA target site by PCR. Half of the PCR product was digested with NcoI. By comparing the ratio of the digested product with an intact restriction site (lower band) to the non‐digested product containing a mutated restriction site (upper band) after the addition of NcoI (+) gives an indication of the efficiency of the induction of mutations.CFrogs injected with the CRISPR/Cas9 system and raised to adulthood. The F1 embryos were sequenced for mutations in *foxm1*. Four frameshift mutations were identified.DGenotypes used in this study.ETadpoles from a *foxm1* ^+/−^ cross were raised to NF50, amputated and the tails collected at 3dpa for RNA expression and the heads for genotyping. *Foxm1* expression was analysed by qPCR, using *ef1α* as a reference (*n* = 3 with at least 3 embryos per sample). The data are expressed as the mean ± SD.FA third of the tails of *foxm1* knockdown (Crispr mosaic F0) and wt tadpoles at NF50 were amputated and the tadpoles left to regenerate for 9 days. The images show representative tails at 9dpa.G, HTo quantify the rate of regeneration, the ratio of the length of the regenerate to the length that has originally be amputated was compared for the spinal cord (G) and the whole tail (H). The graph represents the mean ± SD of three independent experiments with at least five tadpoles in each experiment. Data presentation: For testing statistical significance, an unpaired *t*‐test was used in E and a two‐way ANOVA followed by a Sidak multiple comparison test in G and H. *P < 0.05 and ****P < 0.0001.

**Figure EV3:**
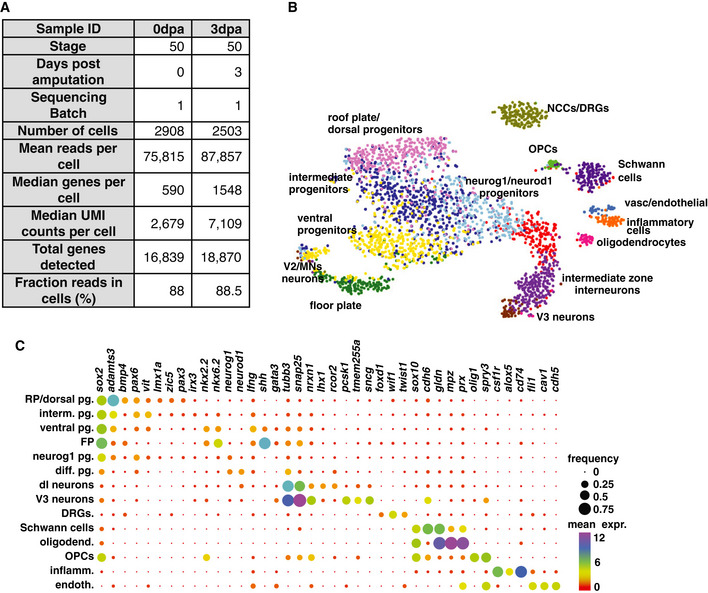
Characterisation of the *Xenopus* spinal cord by single‐cell RNA sequencing AMetadata of the scRNA‐seq experiment.Bt‐SNE representation of the dataset from 0 dpa with the different cell types identified using a dynamic tree cut algorithm.CBubble plot representing the proportion of cells (size of the dot) and level of expression (colour of the dot) for the genes used to identify the cell types in (B).

**Figure EV4:**
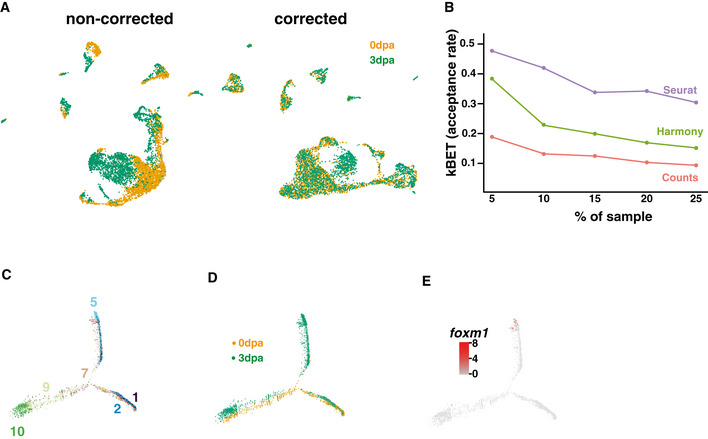
Characterisation of the *foxm1* positive cells AUMAP representation of the scRNA‐seq dataset before (left panel) and after (right panel) batch correction using Seurat.BUnbiased acceptance rate at the indicated subsampling percentile in the raw data (Counts) and after batch correction using Harmony or Seurat algorithm.CUnsupervised pseudo‐time of the whole scRNA‐seq dataset. The distribution of the different clusters along the pseudo‐time is indicated with the colours and numbers as described in Fig 3F.DPseudo‐time of the whole scRNA‐seq dataset with cells from 0 dpa in orange and from 3 dpa in green.EPseudo‐time representation showing the cells expressing *foxm1* (red dots).

**Figure EV5:**
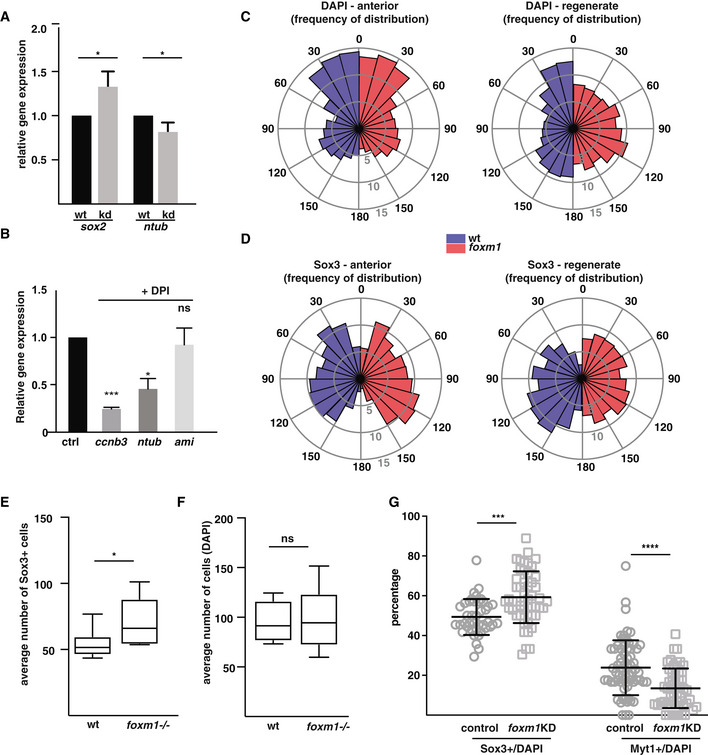
Effect of impairing *foxm1* expression on the organisation of the regenerating spinal cord ATails from tadpoles *foxm1* knockdown (mosaic Crispr F0, kd) and control (wt) NF50 tadpoles were amputated and left to regrow for 3 days. RNA was isolated from the regenerates and expression levels of *sox2* and *ntubulin* analysed by qPCR using *ef1*α as a reference. *sox2*: *n* = 4, *ntubulin*: *n* = 6, with at least 20 tails per sample,BEffect of DPI treatment on the expression of known transcriptional targets of Foxm. Embryos were treated with DPI as described in Fig EV1F, and the expression of *ccnb3* (a Foxm1 target gene)*, ntub* (a marker of differentiated neurons) and *ami* (a gene expressed in endothelial cells) was analysed by RT–qPCR using *ef1α* as control.C, DRose plot histograms showing the percentage frequency distribution of the angles of DAPI^+^ nuclei (C) or Sox3^+^ nuclei (D) in wt (blue) or *foxm1* ^−/−^ spinal cords (red). Angles are distributed into 12 bins from 0 to 180 degrees using a MATLAB script. Dorsal = 0 degrees, lateral = 90 degrees and ventral = 180 degrees. The inner, middle and outer circle corresponds to 5, 10 and 15%, respectively. Ten sections from *n* = 4 animals were analysed per genotype. Total cell counts were as follows: wt anterior (DAPI^+^ 2166; Sox3^+^, 701), *foxm1* ^−/−^ anterior (DAPI^+^, 2246; Sox3^+^, 707), wt regenerate (DAPI^+^, 1927 nuclei; Sox3^+^, 826) and *foxm1* ^−/−^ regenerate (DAPI^+^, 2393; Sox3^+^, 1125). *P* < 0.0001 for regenerate spinal cords and *P* > 0.05 for anterior spinal cords as analysed by Kolmogorov–Smirnov tests.E, FQuantification of the absolute number of cells per section expressing Sox3 (E) and nuclei (DAPI, F) in the regenerate of wild type (wt) and *foxm1* ^−/−^ knockout tadpoles at 5 dpa. The number is derived from the same sections analysed in Fig 5C, and the quantification is derived from the analysis of 8 tadpoles with an average of 15 sections per tail.GThe tails of control and *foxm1*KD animals, fixed at 5 days post‐amputation, were sectioned and labelled with the Sox3 or Myt1 antibody followed by DAPI staining. The ratio of Sox3 and Myt1 per number of DAPI stained nuclei in the spinal cord was quantified and compared between control and foxm1KD tadpoles. Sox3 wt *n* = 6 with 41 sections, CRISPR/Cas9 *n* = 5 with 50 sections, Myt1 wt = 8 with 67 sections and foxm1KD *n* = 7 with 53 sections. Data information: In A and B, the graph represents the mean ± SD of three independent experiments normalised to wt. In E and F, the central line represents the median, the box the 25^th^/75^th^ percentile and the whiskers the min and max values. In G, the graph shows the mean ± SD. For A, E, F and G, the significance was tested with an unpaired *t*‐test, and for B, a one‐way ANOVA with a Tukey post hoc test was used. ns: non‐significant, *P < 0.05, ***P < 0.001 and ****P < 0.0001.

We compared the transcriptome of isolated spinal cords at 1 day post‐amputation (1 dpa) and 3 dpa to spinal cords from intact tails (0 dpa, Fig 1A). Principal component plot, dendrogram of sample‐to‐sample distances and MA plot of the log2 fold change (FC) of expression in relation to the average count confirmed the quality of the data (Fig EV1, EV2, EV3, EV4, EV5). Between 0 dpa and 1 dpa, 2447 differentially expressed (DE) transcripts (|log2(FC)|> 1 and FDR < 0.01) were identified (1,125 down‐ and 1,322 upregulated). Between 0 dpa and 3 dpa, 5,383 genes are differentially expressed (2,746 down‐ and 2,637 upregulated, Fig EV1E, Dataset EV1).

To identify the most enriched biological processes by gene ontology (GO), a non‐biased hierarchical clustering for all DE genes was performed (Fig 1B). We observed three phases: first an increase in expression of genes involved in metabolic processes (cluster I), then a strong upregulation of genes associated with cell cycle regulation (clusters II and III) and finally, a downregulation of expression of genes involved in nervous system development (clusters IV and V, Fig 1B).

Using Ingenuity Pathway Analysis (IPA), we identified potential upstream regulators that could explain changes in expression of downstream target genes, with Foxm1 showing the highest significance at 3 dpa (Fig 1C). Using published RNA‐seq of tail regeneration in *X*. *tropicalis* (Chang *et al*, 2017), we compared changes in expression of known Foxm1 target genes between 0 and 3 dpa in whole tail (WT_d0d3), 0 and 1 dpa (SC_d0d1) and 0 and 3 dpa (SC_d0d3) in spinal cord. *Foxm1* and its transcriptional targets are significantly upregulated only in the spinal cord at 3 dpa, but not in the whole tail (Fig 1D).

We wanted to confirm the expression of *foxm1* during regeneration by *in situ* hybridisation (ISH) and RT–qPCR. ISH shows that *foxm1* is not expressed in the spinal cord at 0 and 1 dpa but is restricted to the regenerating spinal cord at 3 dpa (Fig 1E). The whole‐mount ISH was then sectioned to confirm that *foxm1* is expressed specifically in the regenerating spinal cord (Fig 1E). We then performed RT–qPCR for *foxm1* over a period of 7 days, and its expression peaks at 3 dpa and decreases back to baseline levels at 7 dpa (Fig 1F).

We next wanted to identify the upstream signal(s) regulating its expression. As *foxm1* expression starts at 3 dpa, it is not a direct response to the injury. We tested whether signalling pathways required for tail regeneration promote *foxm1* expression at 3 dpa. A sustained increase of reactive oxygen species (ROS) in the tail is required for its regeneration (Love *et al*, 2013). ROS levels were decreased following amputation using DPI, an inhibitor of the NADPH oxidases (NOX). In NF50 tadpoles treated with DPI from 36hpa until 72hpa, *foxm1* expression decreases by 69% (*P* = 0.032) compared to DMSO controls (Fig EV1F and G). ROS are upstream of different signalling pathways, including FGF (Lin & Slack, 2008; Love *et al*, 2013). Furthermore, Sonic hedgehog (Shh) signalling is also required for tail regeneration (Beck *et al*, 2003; Hamilton *et al*, 2021) and induces *Foxm1* expression in the developing cerebellar granule neuron precursors (Schüller *et al*, 2007). However, *foxm1* expression is not affected by treating amputated tails treated with an FGF receptor kinase inhibitor (SU5402, Fig EV1H) or a Shh signalling inhibitor (cyclopamine, Fig EV1I).

### Foxm1 is required for spinal cord regeneration

To test the role of Foxm1 during regeneration, we designed a guide RNA (gRNA) targeted at bases 129–152 downstream of the ATG to knock down and knock out *foxm1* expression using CRISPR/Cas9 (Fig EV2A). The efficacy of the gRNA was assessed by restriction fragment length polymorphism analysis (RFLP). Co‐injection of the gRNA with *cas9* mRNA did not lead to the destruction of the NcoI site but co‐injection with 0.6 and 1.5 ng of Cas9 protein leads to NcoI‐resistant PCR products in a dose‐dependent fashion (Fig EV2B). Sequencing of individual clones revealed that indels occur in 50–90% of the *foxm1* locus. We have identified four frameshift mutations that were germline transmitted and F0 frogs with these mutations were backcrossed with wild types to establish *foxm1* ^+/−^ lines used to generate *foxm1* ^−/−^ tadpoles (Fig EV2C and D). We then confirmed these mutations lead to a decrease in *foxm1* expression by RT–qPCR, with homozygotes mutants displaying an 80% reduction in *foxm1* expression compared to wild type (*P* < 0.0001, Fig EV2E).

**Figure 2:**
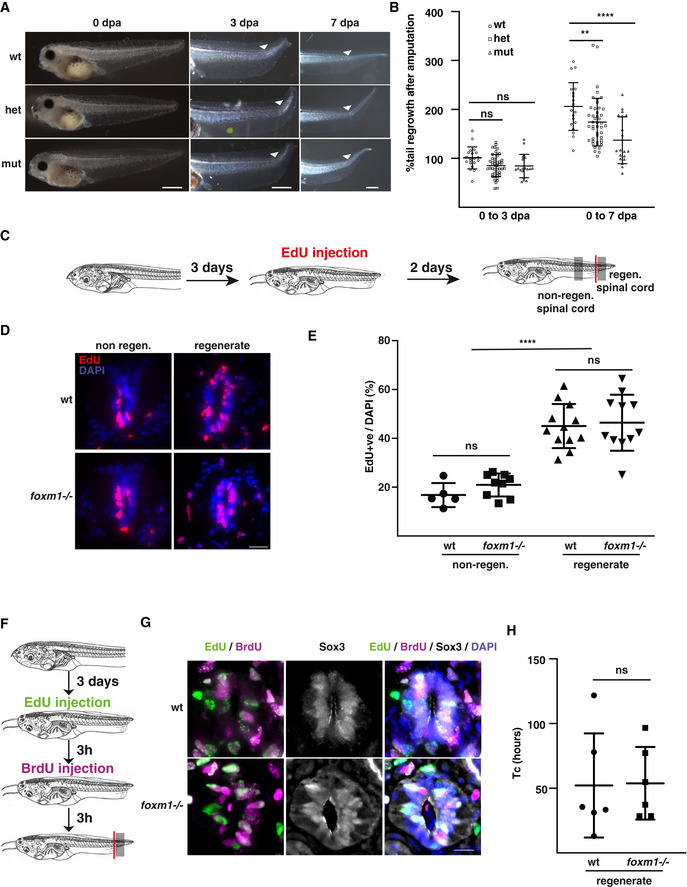
Foxm1 is required for spinal cord regeneration but does not regulate the length of the cell cycle ANF40 tadpoles with the following genotypes *foxm1* ^−/−^ (mut), *foxm1* ^+/−^ (het) and *foxm1* ^+/+^ (wt) were amputated and left to regenerate for 7 days. The images show representative tails at 3 and 7 dpa. The white arrowheads indicate the amputation site.BQuantification of the rate of regeneration. The ratio of the length of the tail regenerate at 3 and 7 dpa was compared to the length of the tail originally amputated at 0 dpa. The graph represents the mean ± SD of five independent experiments from three different clutches with at least five tadpoles in each experiment.CExperimental setup for EdU labelling, *foxm1* knockout and wt tadpoles were amputated and left to regenerate for 3 days. Tadpoles were then injected with EdU and 2 days later the tails were fixed, sectioned and stained for EdU and DAPI.DRepresentative images of EdU (red) and DAPI (blue) staining at 5 dpa.EThe graph represents the mean ± SD of EdU^+^ cells over the total number of cells in the spinal cord of 5–12 tadpoles. Each data point represents a tadpole, with an average of 9 sections analysed per animal.FExperimental setup for Dual‐Pulse S‐phase Labelling: NF50 tadpoles at 3 dpa were injected with EdU, and 3 h later, the same tadpoles were injected with BrdU. Six hours after the first injection, the tails were fixed, sectioned and labelled for Sox3, Edu, BrdU and DAPI.GRepresentative images of EdU (green), BrdU (magenta), Sox3 (white) and DAPI (blue) staining at 3 dpa.HQuantification of images in (G). The graph represents the mean ± SD of 6 tadpoles with an average of 13 sections per tadpole analysed. Data information: Two‐way ANOVA with Tukey’s post hoc tests was used for B and E and an unpaired *t*‐test for H. ns: non‐significant, **P < 0.01 and ****P < 0.001. Scale bar is 500 µm in A and 25 µm in D and G.

We then analysed the ability of NF40 tadpoles to regenerate their spinal cord depending on their genotype (Fig 2A and B). About 40% of the tails were removed and the ratio of regeneration was determined between 0 to 3 and 0 to 7 dpa by dividing the length of the regenerate by the length of the amputated tail. No differences were observed for 0 to 3 dpa, but for 0 to 7 dpa the rate of regeneration was on average 35% lower (*P* < 0.0001) compared to controls (Fig 2B). Interestingly, *foxm1* ^+/−^ tadpoles have an intermediate phenotype (reduction of 17%, *P* = 0.0055), suggesting a dose‐dependent effect of *foxm1* expression on appendage regeneration. The same effect was observed in F0 mosaic tadpoles at NF50, suggesting that the impairment in spinal cord regeneration is not stage or mutation specific (Fig EV2, EV3, EV4, EV5).

Could the impaired regeneration be caused by defective proliferation? To determine the rate of proliferation in the regenerating spinal cord, wt and *foxm1* ^−/−^ tadpoles were injected with EdU at 3 dpa, followed by a 2‐day chase (Fig 2C–E). As expected, we observed a higher proportion of EdU^+^ cells in the regenerate than in the non‐regenerating spinal cord (˜45 and ˜20%, respectively). However, no difference in the proportion of EdU^+^ cells between wt and *foxm1* ^−/−^ was observed, suggesting that Foxm1 does not affect the overall length of cell cycle. To confirm these data, we measured the absolute length of the cell cycle by Dual‐Pulse S‐phase Labelling (DPSL; Thuret *et al*, 2015). We first established the growth fraction by quantifying the proportion of cycling progenitors in the regenerating spinal cord (PCNA^+^ Sox3^+^) in wt and mutant tadpoles. In both cases, around 95% of Sox3^+^ cells are also PCNA^+^ (Appendix Fig S1A and B). To establish the absolute length of the cell cycle at 3 dpa, the tadpoles were injected first with EdU, then 3 h later with BrdU and fixed 3 h later (Fig 2F). The total length of the cell cycle (Tc) was about 50 h both in wild type and in *foxm1* ^−/−^ tadpoles, confirming that Foxm1 does not regulate the overall length of the cell cycle during spinal cord regeneration (Fig 2G and H).

As it has been shown that knocking down *foxm1* expression decreases the proliferation of neuronal progenitors during primary neurogenesis in *X. laevis* (Ueno *et al*, 2008), we tested whether this was also the case in *X. tropicalis* (Appendix Fig S2). Embryos at 1‐cell stage were injected either with morpholino control (MOC) or with morpholino targeted to the exon3–intron3 (MOe3i3) and intron3–exon 4 (MOi3e4) splice sites of the *foxm1* mRNA (MOF1). In parallel, we also analysed embryos injected with Cas9/gRNA against *foxm1* (Crispr F1) or Cas9 alone (Crispr C). We first analysed the ability of the morpholino and gRNA/Cas9 to reduce *foxm1* expression by RT–qPCR when injected at the one‐cell stage (solid bars) or in one cell of a 2‐cell stage embryo (hatch bars Appendix Fig S2A). Injection of MOF1 at 1‐cell stage leads to a reduction of ˜70% of *foxm1* expression (˜40% when injected in 1 cell at 2‐cell stage), whilst injection of Crispr F1 causes a reduction of about 55% (and ˜30% when injected in 1 cell at 2‐cell stage, Appendix Fig S2A). We then injected 1 cell at the 2‐cell stage with MOC, MOF1, Crispr F1 or Crispr C together with a rhodamine‐DEXTRAN as a tracer (Appendix Fig S2B). Embryos were fixed at NF13 and stained for phospho‐Histone H3 (pH3). Quantification of pH3 cells in the neural plate shows no difference between injected and non‐injected sides in all conditions, suggesting that Foxm1 does not promote proliferation during primary neurogenesis in *Xenopus tropicalis* (Appendix Fig S2C).

### Organisation of the *Xenopus* spinal cord at stage NF50

To understand the role of Foxm1 during spinal cord regeneration, we wanted to characterise this cell population at the molecular level using single‐cell RNA sequencing (scRNA‐seq). As the cellular organisation of the *Xenopus* spinal cord is not well described, we first used the 10X Genomics platform to sequence 2908 cells from uninjured spinal cord (Fig EV3, EV4, EV5). Twelve clusters were identified comprising the different cell types expected in the spinal cord: roof and floor plate (*bmp4* and *shh,* respectively*)*, dorsal (*lmx1a/zic5),* intermediate (*pax6, vit)* and ventral (*nkx2.2, nkx6.2)* progenitors, neurons (*tubb3, snap25),* oligodendrocytes (*mpz, prx)* and oligodendrocyte progenitor cells (OPCs; *sox2, olig1*). Two clusters correspond to cells present in spinal ganglia: dorsal root ganglia (*wif1* and *twist1*) and Schwann cells (*sox10)*. Finally, we observed a small population of inflammatory (*csf1r, alox5*), vascular and endothelial (*fli1*, *cav1*) cells that may be spinal cord resident cells or a contamination from the dissection. However, no mesodermal or skin contamination was identified (Fig EV3B and C).

### Characterisation of *foxm1* ^+^ cells during regeneration

**Figure 3:**
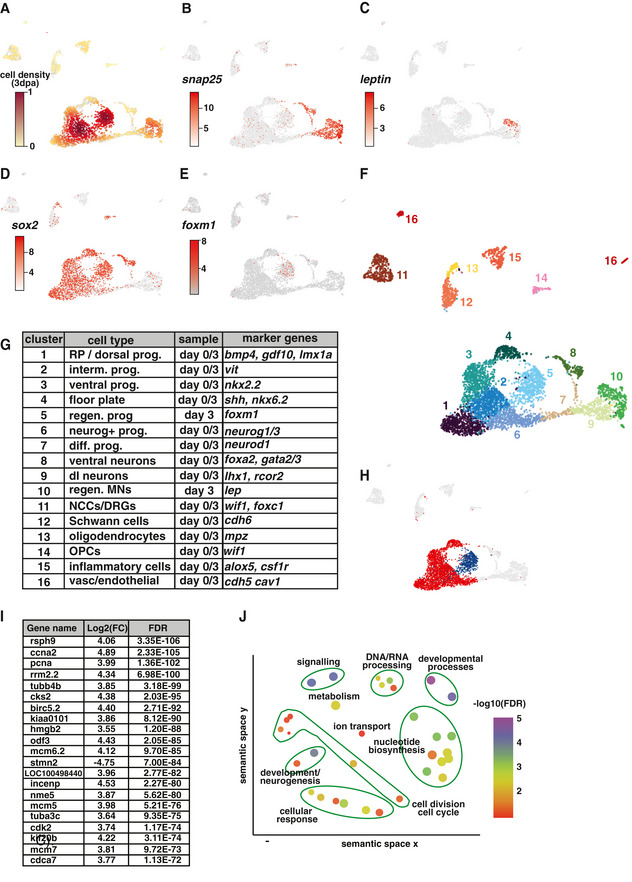
Characterisation of the *foxm1* ^+^ cells in the regenerating spinal cord AUMAP representation of the whole scRNA‐seq dataset (0 dpa and 3 dpa) showing the cell density distribution of the 3 dpa sample.B–EExpression of *snap25* (neuronal marker), *leptin, sox2* (progenitor marker) and *foxm1* on the same UMAP representation as in (A).FUMAP representation of the 16 clusters identified in the scRNA‐seq dataset.GIdentity of the clusters with the most significant differentially expressed gene(s).HSchematic representation of the cells used to identify DE genes and over‐representation of GO terms for cluster 5 (blue cells) corresponding to the *foxm1* ^+^ cluster against the rest of the progenitor cells (red cells).ITwenty most significantly DE genes comparing blue versus red cells ranked by FDR.JGO‐Slim Biological Process terms over‐represented were identified by uploading the DE genes into PANTHER. The GO terms significantly upregulated were then inputted into Revigo (http://revigo.irb.hr/) to generate a plot representation.

We then analysed the changes in the transcriptome during regeneration by combining both 0 dpa and 3 dpa scRNA‐seq datasets (Figs 3 and EV4A and B). We noticed that whilst the different cell types were clustering together, there was a clear shift between cells from 0 and 3 dpa possibly due to a batch effect (Fig EV4A, left panel). To correct for this technical bias, we assessed the two widely used algorithms for batch correction, Harmony and Seurat, using the *k*‐nearest‐neighbour batch‐effect test (kBET) (Butler *et al*, 2018; Büttner *et al*, 2019; Korsunsky *et al*, 2019). Whilst both algorithms improve acceptance rate compared to the non‐corrected data, Seurat outperformed Harmony at all subsampling rates (Fig EV4B). After batch correction, most cells from 0 and 3 dpa aligned in the same space on a UMAP representation with the exception of two areas (Figs EV4A and 3A) corresponding to *lep* ^+^ neurons (*snap25* ^+^ Fig 3B and C) and *foxm1+ve* progenitors (*sox2* ^+^ Fig 3D and E).

We next assigned a cell type identity for each cluster based on the most significant differentially expressed genes (Dataset EV2, Fig 3F and G). The same clusters as 0 dpa were identified with the addition of the two 3 dpa‐specific clusters (*lep* ^+^ and *foxm1* ^+^ clusters). We then performed pseudo‐time analysis at both timepoints to explore the origin of *foxm1* ^+^ cells (Fig EV4, EV5, Trapnell *et al*, 2014). Unsupervised pseudo‐time using the Monocle algorithm places the root on progenitor clusters (Fig EV4C) with one branch terminating with differentiated neurons. However, a 3 dpa‐specific branch was identified with *foxm1* ^+^ cells clustering at the end of this branch (Fig EV4D and E).

To characterise the *foxm1* ^+^ cells, we identified DE genes between the *foxm1* ^+^ cluster and the rest of progenitors (*sox2* ^+^, Fig 3H). The list of the 20 top DE genes ranked by false discovery rate (FDR) shows that the majority are upregulated and many are linked to the cell cycle (*ccna2*, *pcna*, *cdk2*, Fig 3I)). We then identified the GO terms that were significantly over‐represented with PANTHER and used Revigo to generate a plot representation (Fig 3J). Whilst some GO terms are associated with development, neurogenesis and signalling, the majority of the GO terms identified are linked to cell cycle dynamics.

### Cell cycle dynamics during regeneration

**Figure 4:**
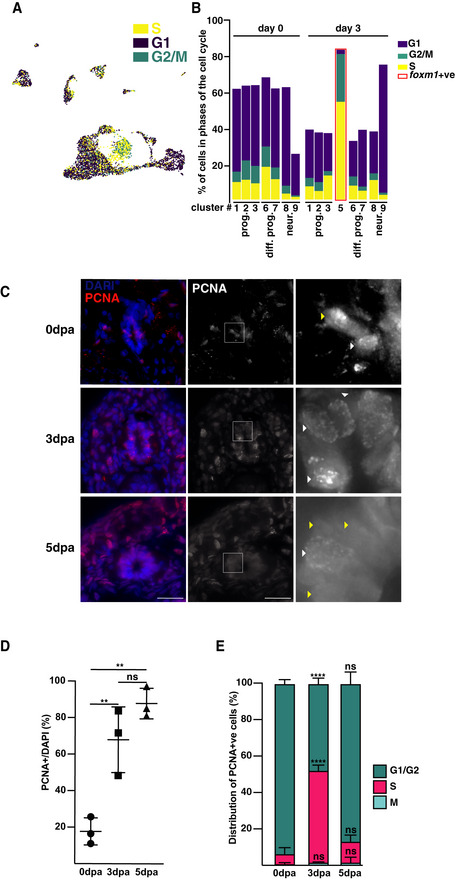
Changes in cell cycle dynamics during regeneration AUMAP projection with inferred cell cycle phase for each cell.BBar plot showing the proportion of cells in G1, G2/M and S phases of clusters of progenitor cells (prog., expressing *sox2* and *sox3*) and differentiating progenitors (diff. prog., expressing *neurod1, neurog1*) and neurons (neur., expressing *snap25*). The total does not always amount to 100% as some clusters have cells from both 0 and 3 dpa. The bar boxes in red represent the *foxm1* positive cluster, and the cluster numbering refers to the cluster identity defined in Fig 3G.CRepresentative sections labelled with an anti‐PCNA antibody (red) and DAPI (blue) at the indicated day after amputation (dpa). The white arrowheads point to cells in S phase in the spinal cord and the yellow arrowhead at cells in G1, G2 and M phases. The right panels correspond to the inset indicated as a white box in the middle panels.DThe ratio of PCNA^+^ per total number of cells (DAPI) in the spinal cord was determined at the indicated times after amputation (*n* = 3, with a mean of 14 sections per data point).EThe PCNA^+^ cells were then distributed in G1/G2 (diffuse signal), S (punctated signal) or M phase (condensed chromatin) at the indicated stage of regeneration. Data information: In D‐E, Data are the mean ± SD of three independent experiments, and one‐way ANOVA with Tukey’s post hoc tests was used. ns: non‐significant, **P < 0.01 and ****P < 0.0001. In C, scale bar represents 25 µm for the left and middle panels and 5 µm for the right panels.

The result of our GO analysis led us to analyse changes in cell cycle dynamics between 0 and 3 dpa (Fig 4). We first used computational inference of cell cycle state on our scRNA‐seq dataset (Fig 4A and B, Aztekin *et al*, 2019). Whilst clusters representing neurons are mainly in G1/G0 both at 0 and at 3 dpa, this proportion decreases in differentiating neurons and progenitor clusters have the highest proportion of cells in G2/M and S phases. The small number of cells in G2, M and S phases in neuronal clusters is probably due to some *sox2* ^+^ cells present in these clusters (Fig 3D). Interestingly, more than 50% of the cells in the *foxm1* ^+^ cluster appeared to be in S phase.

To confirm these changes in cell cycle dynamics, we performed PCNA staining on sections at 0, 3 and 5 dpa (Fig 4C–E). The percentage of PCNA^+^ cells in the spinal cord increases sharply at 3 dpa when compared to 0 dpa (from 18 to 68%) and remains high until 5 dpa (88%; Fig 4D). To estimate the proportion of cells in different phases of the cell cycle, we used the fact that PCNA expression is punctate in S phase and diffuse in G1/G2 phase (Celis *et al*, 1986; Rottach *et al*, 2008) and the chromatin is condensed in M phase (Fig 4C). We observed a transient increase of the proportion of the cells in S phase from 6.6% at 0 dpa to 45.7% at 3 dpa with a return to baseline by 5 dpa (12%; Fig 4E). These data confirmed our scRNA‐seq experiments and suggest that proliferative cells in the spinal cord may have a long S phase in early regeneration.

### Foxm1 regulates the fate of dividing progenitors during regeneration

Because Foxm1 promotes neuronal differentiation in early *Xenopus laevis* development (Ueno *et al*, 2008), we analysed the relative proportions of progenitors and neurons in the regenerating spinal cord in wt and *foxm1* ^−/−^ tadpoles. Expression analysis by RT–qPCR shows an 33% increase (*P* = 0.02) in *sox2* and a 18% (*P* = 0.01) decrease in *ntub* expression in F0 tadpoles with knocked‐down *foxm1* expression (Fig EV5A). Interestingly, reducing ROS levels with DPI also impairs expression of *ntub* and *ccnb3,* a well‐characterised Foxm1 transcriptional target. However, DPI treatment had no effect on the expression of *ami*, a gene expressed in endothelial cells (Fig EV5B).

**Figure 5:**
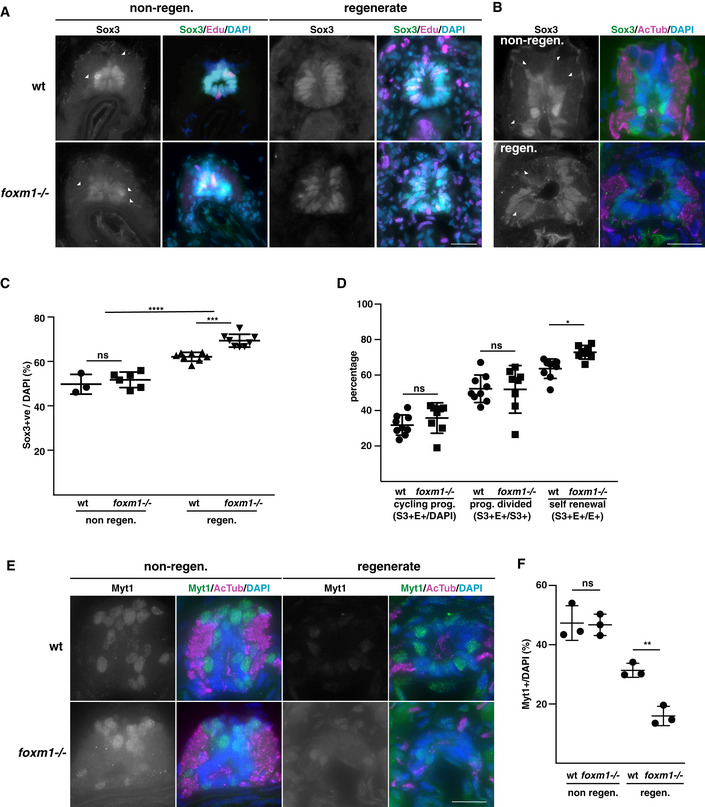
Foxm1 promotes the differentiation of neural progenitors in the regenerating spinal cord ARepresentative sections of spinal cords from tadpoles injected with EdU at 3 dpa and fixed at 5 dpa. After sectioning, the samples were labelled with antibodies against Sox3 (green), EdU (magenta) and DAPI (blue). White arrowheads show Sox3 positive extensions.BSections of spinal cords at 5 dpa in the stump (non‐regen.) or in the regenerate (regen) labelled with anti‐Sox3 (green), anti‐Acetylated Tubulin (AcTub, magenta) and DAPI (blue). White arrowheads show Sox3 positive extensions.CQuantification of the images shown in (A). The ratio of Sox3^+^ per total number of cells (DAPI) in the spinal cord was determined in the stump (non‐regen.) and the regenerating spinal cord (regen.) in wt and *foxm1* ^−/−^ tadpoles (*n* = 3–8, with an average of 15 sections per data point).DQuantification of the proportion of cycling progenitors (Sox3^+^EdU^+^/DAPI, S3^+^E^+^/DAPI), the proportion of progenitors having divided (Sox3^+^EdU^+^/Sox3^+^, S3^+^E^+^/S3^+^) and the proportion of progenitors self‐renewal (Sox3^+^EdU^+^/EdU^+^, S3^+^E^+^/E^+^) in the regenerate. The graph represents the mean ± SD from 9 tadpoles (wt) and 8 tadpoles (*foxm1* ^−/−^) with an average of 18 sections per data point.ERepresentative sections of spinal cords from tadpoles injected with EdU at 3 dpa and fixed at 5 dpa. After sectioning, the samples were labelled with antibodies against Myt1 (green), EdU (magenta) and DAPI (blue).FQuantification of images shown in (E). The graph represents the mean ± SD from 3 tadpoles with an average of 12 sections each. Data information: For C, D and F, a two‐way‐ANOVA with Tukey’s post hoc tests was used and the graphs represent the mean ± SD. ns = non‐significant, *P < 0.05, **P < 0.01, ***P < 0.001 and ****P < 0.0001. For A, B and E, the scale bar represents 25 µm.

We next analysed Sox3 expression by immunofluorescence using anti‐Sox3 antibodies in the non‐regenerating and regenerating spinal cord of wt and *foxm1* ^−/−^ NF50 tadpoles at 5 dpa. As expected, in the non‐regenerating spinal cord Sox3 is expressed in the cells lining the ventricle and we do not observe major differences between wild type and *foxm1* ^−/−^ (Fig 5A and C). We also observe Sox3^+^ staining outside of the progenitor zone of the spinal cord (Fig 5A and B, white arrowheads). We confirmed that these Sox3 projections expand to the mantle zone of the spinal cord by co‐staining with an anti‐acetylated Tubulin antibody (Fig 5B). In the wt regenerate, the spinal cord appears as an almost monolayer of cells around the central canal. Sox3 is expressed only in cells of the lateral spinal cord. These data reveal that the regenerating spinal cord conserved some cellular organisation (Fig 5A). In contrast, in *foxm1* ^−/−^ tadpoles, we observed multiple cell layers and Sox3 expression is expressed more broadly (Fig 5A). To assess the distribution of cells in wt and *foxm1* ^−/−^ spinal cords, the angle of all nuclei (DAPI, Fig EV5C) and Sox3^+^ nuclei (Fig EV5D) relative to the centre of the central canal was calculated. Whilst in the wt regenerating spinal cords, there are less cells laterally, this is not the case in *foxm1* ^−/−^ tadpoles (Fig EV5C). Furthermore, we observed a greater proportion of Sox3^+^ cells both dorsally and ventrally in the mutant compared to wt (Fig EV5D). Interestingly, no difference was observed in the distribution of DAPI and Sox3 in the anterior spinal cord (non‐regen. Figure EV5C and D). Taken together, these data may explain why the spinal cord does not elongate in *foxm1* mutants, whilst the rate of proliferation is unchanged.

The proportion of Sox3^+^ cells in the non‐regenerating spinal cord is about 50% in wt and *foxm1* ^−/−^ tadpoles. In contrast, the proportion of progenitors increased in the regenerating spinal cord from 62.1 ± 3.5% in wt to 69.4 ± 2.9% in *foxm1* ^−/−^ (*P* < 0.001, Fig 5C). Importantly, this increase in the proportion of Sox3^+^ cells is not due to an overall change in the number of cells in the regenerate. When considering absolute number of Sox3^+^ cells, we observe a significant increase in *foxm1* ^−/−^ compared to wild type (Fig EV5E), whilst the average number of cells (quantified using DAPI) is unchanged (Fig EV5F). Furthermore, very little apoptosis is observed in the spinal cord at this stage and no significant difference in the proportion of apoptotic cells between wt and *foxm1* ^−/−^ regenerate was detected (Appendix Fig S3A–C).

Because we estimated the cell cycle to be approximately 2 days (Fig 2H), we labelled cycling cells with EdU at 3 dpa and determined their fate after one cell cycle at 5 dpa using immunofluorescence. We first quantified the proportion of dividing progenitors over the total number of cells (S3^+^E^+^/DAPI) or over the total number of progenitors (S3^+^E^+^/S3^+^, Fig 5D) in tadpoles following amputation. Both ratios are similar in wt and *foxm1* ^−/−^ tadpoles, indicating that knocking out *foxm1* does not alter the rate of progenitor division. In contrast, the proportion of self‐renewal (S3^+^E^+^/E^+^) increases significantly in the *foxm1* ^−/−^ compared to wt tadpoles (from 65 to 75%, *P* = 0.006, Fig 5D). The stable rate of proliferation of progenitors combined with the increased rate in self‐renewal suggests that there is a shift in the fate of dividing progenitors from differentiation towards self‐renewal.

To confirm these data, we analysed the expression of Myt1 as a neuronal marker in wt and *foxm1* ^−/−^ NF50 tadpoles at 5 dpa (Fig 5E and F). Knocking out *foxm1* does not affect the percentage of Myt1^+^ cells in the non‐regenerating spinal cord (˜43% of spinal cord cells). However, in the regenerate we observed a sharp reduction of Myt1^+^ cells in the mutant compared to wt (30% versus 14%, *P* < 0.01). Similar data were obtained when we analysed mosaic F0 injected with gRNA targeting the *foxm1* locus compared to Cas9‐injected controls (Fig EV5G). Thus, in *foxm1* mutants, there is an increase of progenitors at the expense of differentiated neurons. Rather than promoting proliferation, Foxm1 affects cell fate by promoting differentiation.

## Discussion

**Figure 6:**
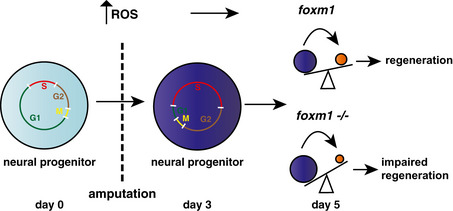
Model of neural progenitor cell behaviour during spinal cord regeneration in wild type (wt) and *foxm1* ^−/−^ tadpoles The blue circles correspond to *sox2/3*+ve cells (progenitors) and the orange circle to *snap25* ^+^ cells (neurons).

Using a combination of bulk and single‐cell RNA‐seq experiments on isolated spinal cords during tail regeneration in *X*. *tropicalis*, we identified a new population of cells present exclusively in the regenerating spinal cord. It is characterised by the expression of *foxm1,* and GO analysis reveals that genes involved in cell cycle, metabolism and neurogenesis are over‐represented. Knocking out the *foxm1* gene impairs spinal cord regeneration and alters the fate of the dividing progenitors with an increase in self‐renewal division and a decrease in the production of neurons (Fig 6).

To characterise the *Xenopus* spinal cord at the molecular level, we undertook a single‐cell RNA‐seq approach. The number of cells and the depth of sequencing did not allow us to unambiguously determine the identity of progenitors and neurons subtypes but we identified the main cell types present in a vertebrate spinal cord, such as roof plate, dorsal and ventral progenitors, neurons and oligodendrocytes. Comparison of their transcriptomes at 0 and 3 dpa reveals that only two clusters are 3 dpa‐specific: a cluster of progenitors characterised by *foxm1* expression and a cluster of differentiated neurons characterised by *leptin* and *leptin receptor* expression. These neurons have been identified previously but whether they are located in the regenerate and their role(s) during regeneration is still unclear (Aztekin *et al*, 2019; Kakebeen *et al*, 2020).

Foxm1 has been shown to have a role in neuronal differentiation during primary neurogenesis in *Xenopus laevis* (Ueno *et al*, 2008) and in the mouse telencephalon (Wu *et al*, 2014). However, in both cases, the role of Foxm1 seems to be dependent on its ability to control the overall length of the cell cycle. By contrast, in *Xenopus tropicalis* we do not observe a difference in proliferation during primary neurogenesis and in the regenerating spinal cord in *foxm1* ^−/−^ compared to controls. Furthermore, the total length of the cell cycle of neural progenitors in the regenerating spinal cord is not affected by Foxm1 expression. These data suggest a cell cycle independent role for Foxm1 during this process. Cell cycle regulators such as Cdc25b and Ccnd1 have been shown to play a role during neuronal development independently of their primary function (Lukaszewicza & Anderson, 2011; Hydbring *et al*, 2016; Bonnet *et al*, 2018). Thus, it is possible that Foxm1 can promote neuronal differentiation in the regenerating spinal cord without affecting the overall length of the cell cycle.

Whilst we do not observe differences in the rate of proliferation in mutant and wt tadpoles, a striking characteristic of the *foxm1* ^+^ cluster is the changes in the proportion of cells in each phase of the cell cycle compared to *foxm1* ^−^ progenitors. About 50% of the *foxm1* ^+^ cells are in S phase and 40% in G2/M, leaving only about 10% of cells in G1. Interestingly, similar changes in cell cycle have been observed in Axolotls, suggesting that changes in cell cycle dynamics may be a general principle of spinal cord regeneration (Rodrigo Albors *et al*, 2015; Cura Costa *et al*, 2021). The high proportion of cells in S phase could be due to a synchronised cell cycle as suggested in Axolotl (Cura Costa *et al*, 2021) or an extension of the relative length of S phase. It has been suggested that a long S phase may be necessary for progenitors undergoing self‐renewal division to ensure genome integrity, especially in response to high level of ROS present in the nervous system (Arai *et al*, 2011; Narciso *et al*, 2016; Turrero García *et al*, 2016). The regenerating tail is an oxidative environment and we show here that *foxm1* requires ROS for its expression. Furthermore, Foxm1 has been shown to ensure chromosomal stability and genome integrity in U2OS and aged fibroblasts (Laoukili *et al*, 2005; Macedo *et al*, 2018), raising the intriguing possibility that Foxm1 might ensure that the expansion of the progenitor pool does not lead to genomic instability during regeneration.

The expansion of the neural stem cell pool is required for spinal cord regeneration in axolotl, zebrafish and *Xenopus* (Ogai *et al*, 2014; Muñoz *et al*, 2015; Rodrigo Albors *et al*, 2015). Here, we show that regeneration also requires the precise control of neuronal differentiation (Fig 6). In mammals, ependymal cells also re‐enter the cell cycle upon spinal cord injury (SCI) and are able to self‐renew but differentiate mainly into astrocytes (Meletis *et al*, 2008). Recent evidence suggests increased neuronal differentiation improves the recovery of function following SCI in mice (Fukuoka *et al*, 2021). Our data show that Foxm1 has an important role in promoting NPCs to differentiate into neurons during regeneration. Uncovering the signals and regulatory networks that allow Foxm1 to drive neuronal differentiation may open new opportunity to enhance spinal cord regeneration in species with limited regenerative capabilities.

## Materials and Methods

### *Xenopus tropicalis* growth and tail amputation

*X. tropicalis* embryos were obtained and raised as described (Collu *et al*, 2012). Tail amputation was performed at Nieuwkoop and Faber stages (NF)40‐50 using a scalpel (Nieuwkoop & Faber, 1994). Tadpoles were anaesthetised with 0.01–0.02% MS‐222 (for NF40 and NF50 tadpoles, respectively) in 0.01× Marc’s Modified Ringer (MMR) solution (10 mM NaCl, 0.2 mM KCl, 0.1 mM MgSO_4_, 0.2 mM CaCl_2_, 0.5 mM HEPES, pH 7.4), followed by recovery in 0.01X MMR. All animal procedures complied with the UK Animal (Scientific Procedures) Act 1986 and were conducted with UK Home Office approval.

### RNA sequencing

Bulk sequencing: Twenty spinal cords were isolated and immediately transferred into TRIzol (Life Technologies) for each timepoint in triplicate. Following RNA extraction according to the manufacturer’s instructions, total RNA concentration was quantified using Qubit HS RNA assay kit (Invitrogen) on the Qubit Fluorometer 2.0 (Invitrogen). Integrity was tested with the Agilent RNA 6000 Pico kit on the Agilent Bioanalyser. Samples with an RNA Integrity Number (RIN) ≥ 7 were considered of acceptable quality. RNA‐seq was performed with Illumina NextSeq 500 using unpaired‐end sequencing at the GeneCore facility (EMBL). Adapter sequences were trimmed using Trimmomatic v0.38. After quality control, the reads were converted to a FASTQ format and mapped on the v9.1 of the *X*. *tropicalis* transcriptome using bwa. The number of reads per transcript was determined using HTseq, and the idxstats files were used to identify differentially expressed (DE) genes using the general linear model glmQLFit in DESeq2 with R. DE genes with a |log2(FC)|> 1 and FDR < 0.01 were considered significant. For the clustering, the k‐mean was determined at 5 using the Elbow method and enriched gene ontology (GO) terms were identified using Fidea (D’Andrea *et al*, 2013). The full RNA‐seq dataset was then uploaded onto the Ingenuity Pathway Analysis (IPA, Qiagen) software. Upstream regulatory analysis was performed for DE genes with a |Log2FC|> 1 and FDR < 0.001 (992 genes for 0 dpa/1 dpa and 2720 genes for 0 dpa/3 dpa). This analysis predicts upstream molecules such as transcription factors that may be causing the observed change in gene expression (Krämer *et al*, 2014).

For single‐cell RNA‐seq (scRNA‐seq), ten spinal cords at 0 and 3 dpa were isolated and transferred in Modified Ringer’s (MR, 100 mM NaCl, 1.8 mM KCl, 2 mM CaCl_2_, 1 mM MgCl_2_, 5 mM Hepes pH7) with 20 µM actinomycin D for 10 min to prevent *de novo* synthesis of mRNA (Wu *et al*, 2017). The spinal cords were transferred in CMF‐MR (100 mM NaCl, 1.8 mM KCl, 1 mM EDTA and 5 mM Hepes pH7), cut in small pieces using a fine scapel and incubated in 200 µl of 0.5% Trypsin‐EDTA without dye (GIBCO) for 30 min at 28°C with shaking at 600 rpm. After a 5 min spin at 300 rcf at 4°C, the spinal cords were incubated for 15 min at room temperature (RT) in 180 µl of MR with 0.3 U/µl of DNaseI (D5025, Sigma). After trituration, 20 µl of Collagenase IV at 50 U/µl was added and the samples were incubated 30 min at 28°C with shaking at 600 rpm. The samples were passed through a flame elongated capillary, applied to a 5 µm strainer and collected in MR with 0.0375% BSA. The samples were pelleted by centrifugation at 300 rcf at 4°C for 5 min, washed with MR‐BSA and resuspended in 35 µl of MR‐BSA. The cell number (4.10^5^–5.10^5^ cells/ml) and viability (above 90%) were estimated on a Countess Cell Counter (Invitrogen). The cells were then loaded onto the 10X Genomics platform for processing.

### Single‐cell RNA‐seq analysis

- Building custom genome for mapping: The *X*. *tropicalis* genome v.9.1 was downloaded from Xenbase.org. A number of genes had duplicated entries where the same gene ID was assigned to different gene names. The majority of these genes had an overlapping transcript; we therefore used the GFFReads merging option to merge these transcripts.
- Data pre‐processing: The sequence files from the sequencer were processed using 10x Genomics custom pipeline Cell Ranger v2.2.0. The fastq files were aligned to the custom genome using the default parameters of Cell Ranger. The pipeline identified the barcodes associated with cells and counted UMIs mapped to each cell. Cell Ranger uses STAR aligner to align reads to the genome discarding all the counts mapping to multiple loci. The uniquely mapped UMI counts are reported in a gene by cell count matrix represented as a sparse matrix format. The Cell Ranger’s aggr command was used to aggregate the samples from 0 and 3 dpa whilst keeping the default down‐sampling parameter enabled.
- Filtering: Low‐quality cells were removed from the dataset to ensure that the technical noise did not affect the downstream analysis. Three commonly used parameters were used for cell quality evaluation: the number of UMIs per cell barcode (library size), the number of genes per cell barcode and the proportion of UMIs that are mapped to mitochondrial genes. Cells that have lower UMI counts than three median absolute deviation (MAD) for the first two matrices and cells having higher proportion of reads mapped to mitochondrial genes with a cutoff of four MADs were filtered out.

After this initial filtering, 5,271 cells (2,870 0 dpa and 2,401 3 dpa) out of 5,411 cells remained for downstream analysis. Violin plots for these three metrics were then plotted to identify cells that have outlier distributions which can indicate doublets or multiplets of cells. However, no outliers were identified so no further filtering was done.

- Classification of cell cycle phase: Seurat’s CellCycleScoring method was used to calculate for each cell the score of S phase and G2 M phase based on expression of S and G2/M phase markers. The cell cycle phase of each cell was identified based on the highest positive score of the phases. Cells that are expressing neither of the S phase and G2/M phase genes would have a negative value for both of these phases and were assigned as being in G1 (Aztekin *et al*, 2019).
- Gene filtering and Normalisation: Genes with average UMI counts below 0.01 were filtered out. After this filtering, 11,215 genes were left for downstream analyses. To take into account the effect of variable library size for each cell, raw counts were normalised using the deconvolution‐based method (Lun *et al*, 2016). Counts from many cells are pooled together to circumvent the issue of higher number of zeros that are common in scRNA‐seq data. This pool‐based size factors are then deconvoluted to find the size factor of each cell. These normalised data are then log‐transformed with a pseudo‐count of 1.
- Visualisation and Clustering: The first step for visualisation and clustering is to identify the highly variable genes (HVGs). To do this, we first decomposed the variance of each gene expression values into technical and biological components and identified the genes for which biological components were significantly greater than zero. These genes are called HVG. HVGs were then used to reduce the dimensions of the dataset using PCA. The dimensions of dataset were further reduced to 2D using t ‐SNE, where 1–14 components of the PCA were given as input. The cells were grouped into their putative clusters using the dynamic tree cut method. Dynamic tree cut method identified the branch cutting point of a dendrogram dynamically and combined the advantage of both hierarchical and K‐medoid clustering approach. Pseudo‐time trajectory was performed using the Monocle 2 algorithm (Qiu *et al*, 2017).
- Batch correction. Two approaches were followed to batch correct the data: (i) using the integration strategy in‐built to Seurat (Butler *et al*, 2018) and (ii) using the Harmony method for single‐cell data integration (Korsunsky *et al*, 2019). Batch‐effect quantification and batch correction method selection were conducted in an unbiased way using the k nearest‐neighbour batch‐effect test (kBET, Büttner *et al*, 2019). For Seurat integration, 500 highly variable genes (HVGs) were first detected using the FindVariableFeatures() function set to the vst selection method. The integrated dataset was obtained by subsequently calling the FindintegrationAnchors() and IntegrateData() functions, both set to a total of 30 dimensions to generate a series of anchors across the data provided in separate Seurat objects. For Harmony, a single Seurat object with the combined data for both timepoints was forwarded to the Harmony::RunHarmony() function in R whilst indicating a sample grouping variable. To evaluate the effectiveness of each method, an evaluation approach was performed using the kBET::kBET() function in R as described in (Tran *et al*, 2020).
- Identification of marker genes: To identify the marker gene for a cluster, we compared a given cluster with all other clusters and changes in expression were tested using the sSeq package. We selected the marker genes for the cluster using manual curation of DE genes with an FDR < 0.01. For PANTHER analysis, all DE genes between the *foxm1* ^+^ cluster were uploaded and tested against the pseudo‐bulk of all the genes expressed in the scRNA‐seq experiment.

### Morpholinos, CRISPR/Cas9 microinjection and inhibitor treatment

The following morpholinos (Gene Tools) were used: *foxm1* MO e3i3 5’‐GAAAGgtactgcacgtataatgaga‐3’, *foxm1* MO i3e4 5’‐tttccctttatagCCAAGCA, Ctrl MO 5’‐CCTCTTACCTCAGTTACAATTTATA. Morpholinos were injected at either 1‐ or 2‐cell stage with a combination of 10 ng of *foxm1* e3i3 and 5ng of *foxm1* i3e4 or 15 ng Ctrl MO. The *foxm1* CRISPR gRNA was designed using Crisprdirect (http://crispr.dbcls.jp) with the target sequence 5’‐CCTGAGCAAACCCTTGTCCATGG. The gRNA cloning was carried out according to published protocols using the following primers fwd 5’‐TAggAACTGTCAAGAAGGCGTTCC, rev 5’‐AAACGGAACGCCTTCTTGACAGTT (Jao *et al*, 2013). Eggs were injected with 300 pg gRNA and 600 ng Cas9 mRNA (from pT3TS‐nCas9n, Addgene; Jao *et al*, 2013) or 600 pg/1.5 ng of Cas9 Protein (M0386, NEB). In all experiments, *foxm1* knockdown corresponds to mosaic F0 tadpoles injected with *foxm1* specific gRNA and 1.5 ng of Cas9 protein (NEB). For *foxm1* knockout, the embryos have been generated from F1 or F2 animals with heterozygous *foxm1* mutations.

For chemical inhibitor treatments, tadpole tails were amputated at NF50 and left to recover for 36 h in 0.01X MMR. Then, ROS signalling was inhibited with 4 µM diphenyleneiodonium (DPI, Merck), FGF signalling with 20 µM SU5402 (Calbiochem) and Shh signalling with 2.5 µM of cyclopamine (Merck). At 3 dpa, the regenerates were collected, total RNA extracted and then processed for RT–qPCR.

### Genotyping

Genomic DNA was extracted from embryos or tails removed by amputation by incubation for 3 h at 55°C in 10 mM Tris pH8, 1 mM EDTA, 80 mM KCL, 0.3% NP40, 0.3% Triton X‐100 and 0.2 mg/ml of proteinase K. The samples were subsequently processed for PCR amplication using the following primers: fwd 5’‐CCACTCATACTCAAGAGACGC, rev 5’‐TGTGAGTTTGCTGGAAGTCCTA. The PCR product was subsequently digested with NcoI for restriction fragment length polymorphism (RFLP) analysis. Alternatively, high‐resolution capillary electrophoresis was performed using a QIAxcel Advanced System (QIAgen).

### Isolation of RNA and qPCR

RNA was isolated from total embryos, tail regenerates and isolated spinal cord using TRIzol (Life Technologies) according to manufacturer’s instructions. cDNA was generated using the Reverse Transcriptase AMV kit (Roche) for whole embryos and tails and Sensiscript Reverse Transcription Kit (Qiagen) for isolated spinal cords. qPCR analysis was performed on the StepOnePlus Real‐Time PCR System using SYBR‐Green reagents (Applied Biosystems). Expression was normalised to the expression levels of *ef1a* or *odc,* and expression values were calculated using the ΔΔCt method.

The following primers were used (5’–3’ sequences): *foxm1* fwd 5’‐AAAGAGGAAGAGAGTGCGCC, rev 5’‐TGGCATTTAGCTGCTCCTCC; *cyclinb3* fwd 5’‐CTGCACTTCCACCATCCAATCCA, rev 5’‐CAACTATATGCGGGACAGAGAG; *cdc25b* fwd 5’‐GCCCAAACCCCTCGAGAAGA, rev 5’‐GCCATCGAAGGTGCGTAGCCT; *ntubulin* fwd 5’‐GGCAGTTACCATGGAGACAGT, rev 5’‐GCCTGTGCCACCACCCAGAGA; *sox2* fwd 5’‐CATGATGGAGACCGATCTCA, rev 5’‐CTTACTCTGGTTGGAGCC; and *ef1a* fwd 5’‐GGATGGAACGGTGACAACATGCT, rev 5’‐GCAGGGTAGTTCCGCTGCCAGA. The primers for *ami* and *odc* are described in Nagamori et al (2014).

### Immunofluorescence and in situ hybridisation

The tails were fixed in 4% paraformaldehyde (PFA) or MEMFA (0.1 M MOPS pH7.4, 2 mM EGTA, 1 mM MgSO_4_, 3.7% formaldehyde) and dehydrated in methanol. Rehydrated samples were embedded in 25% fish gelatin / 20% sucrose and cryosectioned at 12 μm thickness. For non‐regenerating spinal cords, sections were taken at least 250 µm anterior to the amputation plane. For the regenerate, only sections posterior to the amputation plane and anterior to the neural ampulla were considered. The following antibodies were used: rabbit anti‐Sox3 (1:500 at 3 dpa or 1:1,000 at 0 and 5 dpa) and anti‐Myt1 (1:750) were a gift from Nancy Papalopulu; mouse anti‐PCNA was used at 1:500 (PC10, Sigma); rabbit anti‐cleaved caspase 3, Asp175 (1:100, 9661, Cell Signalling Technology), rabbit anti‐pH3 (1:100, 06‐570, Sigma), mouse anti‐BrdU (1:300, clone MoBU‐1, B35128, Invitrogen) and anti‐Acetylated Tubulin (1:1,000, clone 6‐11B1, Sigma). Secondary antibodies were goat anti‐rabbit IgG, Alexa Fluor 488 (A‐11008, Invitrogen) and goat anti‐mouse IgG, Alexa Fluor 568 (A‐11004, Invitrogen).

Whole‐mount in situ hybridisation on embryos and tails was performed as previously described (Harland, 1991). The *foxm1* probe was generated from the TGas064p23 clone linearised with ClaI and transcribed using T7 polymerase.

### Cell cycle analyses

For EdU labelling, NF50 tadpoles at 3 dpa were injected with 12.6 nl of 10 mM EdU (Life Technologies) in DMSO and after 2 days, tails were collected and fixed in MEMFA. EdU was detected using Click‐iT EdU Alexa Fluor 594 Imaging Kit (Life Technologies) following the manufacturer’s instructions.

Dual pulse labelling was used to determine the cell cycle length (*Tc*) of Sox3^+^ NPCs within the regenerate (Martynoga *et al*, 2005). Labelling was performed as described in Thuret *et al* (2015) with minor modifications. NF50 tadpoles at 3 dpa were anaesthetised in MS‐222 and injected with 10 mM EdU (Invitrogen). After 3 h (the injection interval, *Texp*), animals were anaesthetised in MS‐222 and injected with 12.6 nl of 10 mM BrdU (Sigma‐Aldrich). Six hours after the first injection, tails were collected and fixed in 4% PFA, and processed for sectioning as described above. For BrdU staining, sections were incubated for 40 min in pre‐warmed 1N HCl held at a constant temperature of 37°C. The sections were then washed thrice in PBSTx and incubated in blocking solution with 10% heat‐treated lamb serum (HTLS) for 1h at RT. EdU labelling was performed as described above using the Click‐iT EdU Alexa Fluor 647 Imaging Kit (Life Technologies).

The following equation was used to calculate Tc:

$$Tc=\frac{\left(\frac{\mathrm{Texp}}{\left(\frac{L\;cells}{S\;cells}\right)}\right)}{\left(\frac{S\;cells}{P\;cells}\right)}$$

where

S cells = Sox3^+^ cells staying in S phase (Sox3^+^EdU^+^BrdU^+^).

L cells = Sox3^+^ cells leaving S phase (Sox3^+^EdU^+^BrdU^−^).

P cells = proliferative Sox3^+^ cells, calculated as follows:

$$P\;cells=(Sox3^{+}\;cells\;\times \;growth\;fraction).$$

The growth fraction was calculated in Appendix Fig S1B.

### Imaging

*Z* stacks were acquired on a Cell Observer Z1 widefield microscope (Zeiss) using a 63× 1.4NA oil immersion objective. Images were deconvolved using ZEN 2.3 software (adjustable deconvolution). The fast iterative algorithm, Poisson (Richardson Lucy) likelihood, 40 iterations or 0.1% quality threshold) or the constrained iterative algorithm (Poisson likelihood, 40 iterations or 0.1% quality threshold) was used. Cell populations were analysed using the cell counter module of Fiji.

### Statistics

For all the experiments apart from the results of the RNA‐seq, scRNA‐seq and experiments shown on Fig EV5C and D, the normality of the distribution of the data was tested using a Shapiro–Wilk test. When two conditions were compared, a two‐tail unpaired *t*‐test was used. If three or more conditions were compared, a one‐way ANOVA followed by a Tukey post hoc test was used. When multiple conditions were compared across different genotype, a two‐way ANOVA followed by a Tukey post hoc test was performed. For Fig EV5C and D, cumulative distributions were assessed using Kolmogorov–Smirnov tests. All the statistical tests were done using Prism 9.

## Author contributions

Conceptualisation, DP and KD; Formal analysis, DP, LSP, CJG‐D, SMB, Methodology, DP, LSP, RT, SMB and KD; Software, LSP, CJG‐D, SMB and KD; Investigation, DP, LSP, RT and KD; Data curation, SMB and CJG‐D; Visualisation, SMB Writing—original draft, KD; Review & editing, DP, LSP, RT, CJG‐D, SMB and KD; Supervision, KD; Funding acquisition, SMB, KD.

## Conflict of interest

The authors declare that they have no conflict of interest.

## Supporting information



Review Process File

Appendix

Expanded View Figures PDF

Dataset EV1

Dataset EV2

## Data Availability

Bulk RNA‐seq data: ArrayExpress at EMBL‐EBI E‐MTAB‐8785 (https://www.ebi.ac.uk/arrayexpress/experiments/E‐MTAB‐8785/). Single‐cell RNA‐seq: ArrayExpress at EMBL‐EBI E‐MTAB‐8839 (https://www.ebi.ac.uk/arrayexpress/experiments/E‐MTAB‐8839/).

## References

[embr202050932-bib-0001] Arai Y, Pulvers JN, Haffner C, Schilling B, Nüsslein I, Calegari F, Huttner WB (2011) Neural stem and progenitor cells shorten S‐phase on commitment to neuron production. Nat Commun 2: 154 21224845 10.1038/ncomms1155 PMC3105305

[embr202050932-bib-0002] Aztekin C, Hiscock TW, Marioni JC, Gurdon JB, Simons BD, Jullien J (2019) Identification of a regeneration‐organizing cell in the *Xenopus* tail. Science 364: 653–658 31097661 10.1126/science.aav9996 PMC6986927

[embr202050932-bib-0003] Beck CW, Christen B, Slack JM (2003) Molecular pathways needed for regeneration of spinal cord and muscle in a vertebrate. Dev Cell 5: 429–439 12967562 10.1016/s1534-5807(03)00233-8

[embr202050932-bib-0004] Bonnet F, Molina A, Roussat M, Azais M, Vialar S, Gautrais J, Pituello F, Agius E (2018) Neurogenic decisions require a cell cycle independent function of the CDC25B phosphatase. Elife 7: e32937 29969095 10.7554/eLife.32937 PMC6051746

[embr202050932-bib-0005] Butler A, Hoffman P, Smibert P, Papalexi E, Satija R (2018) Integrating single‐cell transcriptomic data across different conditions, technologies, and species. Nat Biotechnol 36: 411–420 29608179 10.1038/nbt.4096 PMC6700744

[embr202050932-bib-0006] Büttner M, Miao Z, Wolf FA, Teichmann SA, Theis FJ (2019) A test metric for assessing single‐cell RNA‐seq batch correction. Nat Methods 16: 43–49 30573817 10.1038/s41592-018-0254-1

[embr202050932-bib-0007] Celis JE, Madsen P, Nielsen S, Celis A (1986) Nuclear patterns of cyclin (PCNA) antigen distribution subdivide S‐phase in cultured cells — Some applications of PCNA antibodies. Leuk Res 10: 237–249 2419706 10.1016/0145-2126(86)90021-4

[embr202050932-bib-0008] Chang J, Baker J, Wills A (2017) Transcriptional dynamics of tail regeneration in *Xenopus tropicalis* . Genesis 55: e23015 10.1002/dvg.23015 28095651

[embr202050932-bib-0009] Cheffer A, Tárnok A, Ulrich H (2013) Cell cycle regulation during neurogenesis in the embryonic and adult brain. Stem Cell Rev Rep 9: 794–805 23900682 10.1007/s12015-013-9460-5

[embr202050932-bib-0010] Collu GM, Hidalgo‐Sastre A, Acar A, Bayston L, Gildea C, Leverentz MK, Mills CG, Owens TW, Meurette O, Dorey K*et al* (2012) Dishevelled limits Notch signalling through inhibition of CSL. Development 139: 4405–4415 23132247 10.1242/dev.081885 PMC3509734

[embr202050932-bib-0011] Cura Costa E, Otsuki L, Rodrigo Albors A, Tanaka EM, Chara O (2021) Spatiotemporal control of cell cycle acceleration during axolotl spinal cord regeneration. Elife 10.7554/eLife.55665 PMC8205487 33988504

[embr202050932-bib-0012] D’Andrea D, Grassi L, Mazzapioda M, Tramontano A (2013) FIDEA: a server for the functional interpretation of differential expression analysis. Nucleic Acids Res 41: W84–W88 23754850 10.1093/nar/gkt516 PMC3692084

[embr202050932-bib-0013] Deuchar EM (1975) Regeneration of the tail bud in Xenopus embryos. J Exp Zool 192: 390 10.1002/jez.1401920311 1141840

[embr202050932-bib-0014] Fukuoka T, Kato A, Hirano M, Ohka F, Aoki K, Awaya T, Adilijiang A, Sachi M, Tanahashi K, Yamaguchi J*et al* (2021) Neurod4 converts endogenous neural stem cells to neurons with synaptic formation after spinal cord injury. iScience 24: 102074 33644710 10.1016/j.isci.2021.102074 PMC7889987

[embr202050932-bib-0015] Gargioli C, Slack JMW (2004) Cell lineage tracing during *Xenopus* tail regeneration. Development 131: 2669–2679 15148301 10.1242/dev.01155

[embr202050932-bib-0016] Hamilton AM, Balashova OA, Borodinsky LN (2021) Non‐canonical Hedgehog signaling regulates spinal cord and muscle regeneration in *Xenopus laevis* larvae. Elife 10: e55665 33955353 10.7554/eLife.61804 PMC8137141

[embr202050932-bib-0017] Hardwick LJA, Philpott A (2014) Nervous decision‐making: to divide or differentiate. Trends Genet 30: 254–261 24791612 10.1016/j.tig.2014.04.001 PMC4046230

[embr202050932-bib-0018] Harland RM (1991) In situ hybridization: an improved whole‐mount method for *Xenopus* embryos. Methods Cell Biol 36: 685–695 1811161 10.1016/s0091-679x(08)60307-6

[embr202050932-bib-0019] Hydbring P, Malumbres M, Sicinski P (2016) Non‐canonical functions of cell cycle cyclins and cyclin‐dependent kinases. Nat Rev Mol Cell Biol 17: 280–292 27033256 10.1038/nrm.2016.27 PMC4841706

[embr202050932-bib-0020] Jao L‐E, Wente SR, Chen W (2013) Efficient multiplex biallelic zebrafish genome editing using a CRISPR nuclease system. Proc Natl Acad Sci USA 110: 13904–13909 23918387 10.1073/pnas.1308335110 PMC3752207

[embr202050932-bib-0021] Kakebeen AD, Chitsazan AD, Williams MC, Saunders LM, Wills AE (2020) Chromatin accessibility dynamics and single cell RNA‐Seq reveal new regulators of regeneration in neural progenitors. Elife 9: e52648 32338593 10.7554/eLife.52648 PMC7250574

[embr202050932-bib-0022] Korsunsky I, Millard N, Fan J, Slowikowski K, Zhang F, Wei K, Baglaenko Y, Brenner M, Loh P, Raychaudhuri S (2019) Fast, sensitive and accurate integration of single‐cell data with Harmony. Nat Methods 16: 1289–1296 31740819 10.1038/s41592-019-0619-0 PMC6884693

[embr202050932-bib-0023] Krämer A, Green J, Pollard J, Tugendreich S (2014) Causal analysis approaches in ingenuity pathway analysis. Bioinformatics 30: 523–530 24336805 10.1093/bioinformatics/btt703 PMC3928520

[embr202050932-bib-0024] Laoukili J, Kooistra MRH, Brás A, Kauw J, Kerkhoven RM, Morrison A, Clevers H, Medema RH (2005) FoxM1 is required for execution of the mitotic programme and chromosome stability. Nat Cell Biol 7: 126–136 15654331 10.1038/ncb1217

[embr202050932-bib-0026] Lin G, Slack JM (2008) Requirement for Wnt and FGF signaling in *Xenopus* tadpole tail regeneration. Dev Biol 316: 323–335 18329638 10.1016/j.ydbio.2008.01.032

[embr202050932-bib-0027] Love NR, Chen Y, Bonev B, Gilchrist MJ, Fairclough L, Lea R, Mohun TJ, Paredes R, Zeef LAH, Amaya E (2011) Genome‐wide analysis of gene expression during *Xenopus tropicalis* tadpole tail regeneration. BMC Dev Biol 11: 70 22085734 10.1186/1471-213X-11-70 PMC3247858

[embr202050932-bib-0028] Love NR, Chen Y, Ishibashi S, Kritsiligkou P, Lea R, Koh Y, Gallop JL, Dorey K, Amaya E (2013) Amputation‐induced reactive oxygen species are required for successful *Xenopus* tadpole tail regeneration. Nat Cell Biol 15: 222–228 23314862 10.1038/ncb2659 PMC3728553

[embr202050932-bib-0029] Lukaszewicza AI, Anderson DJ (2011) Cyclin D1 promotes neurogenesis in the developing spinal cord in a cell cycle‐independent manner. Proc Natl Acad Sci USA 108: 11632–11637 21709239 10.1073/pnas.1106230108 PMC3136279

[embr202050932-bib-0030] Lun ATL, Bach K, Marioni JC (2016) Pooling across cells to normalize single‐cell RNA sequencing data with many zero counts. Genome Biol 17: 75 27122128 10.1186/s13059-016-0947-7 PMC4848819

[embr202050932-bib-0031] Macedo JC, Vaz S, Bakker B, Ribeiro R, Bakker PL, Escandell JM, Ferreira MG, Medema R, Foijer F, Logarinho E (2018) FoxM1 repression during human aging leads to mitotic decline and aneuploidy‐driven full senescence. Nat Commun 9: 2834 30026603 10.1038/s41467-018-05258-6 PMC6053425

[embr202050932-bib-0032] Martynoga B, Morrison H, Price DJ, Mason JO (2005) Foxg1 is required for specification of ventral telencephalon and region‐specific regulation of dorsal telencephalic precursor proliferation and apoptosis. Dev Biol 283: 113–127 15893304 10.1016/j.ydbio.2005.04.005

[embr202050932-bib-0033] McDonald JW, Sadowsky C (2002) Spinal‐cord injury. Lancet 359: 417–425 11844532 10.1016/S0140-6736(02)07603-1

[embr202050932-bib-0034] Mchedlishvili L, Epperlein HH, Telzerow A, Tanaka EM (2007) A clonal analysis of neural progenitors during axolotl spinal cord regeneration reveals evidence for both spatially restricted and multipotent progenitors. Development 134: 2083–2093 17507409 10.1242/dev.02852

[embr202050932-bib-0035] Meletis K, Barnabé‐Heider F, Carlén M, Evergren E, Tomilin N, Shupliakov O, Frisén J (2008) Spinal cord injury reveals multilineage differentiation of ependymal cells. PLoS Biol 6: e182 18651793 10.1371/journal.pbio.0060182 PMC2475541

[embr202050932-bib-0036] Muñoz R, Edwards‐Faret G, Moreno M, Zuñiga N, Cline H, Larraín J (2015) Regeneration of *Xenopus laevis* spinal cord requires Sox2/3 expressing cells. Dev Biol 408: 229–243 25797152 10.1016/j.ydbio.2015.03.009 PMC4826040

[embr202050932-bib-0037] Nagamori Y, Roberts S, Maciej M, Dorey K (2014) Activin ligands are required for the re‐activation of Smad2 signalling after neurulation and vascular development in *Xenopus tropicalis* . Int J Dev Biol 58: 783–791 26154320 10.1387/ijdb.140244kd

[embr202050932-bib-0038] Narciso L, Parlanti E, Racaniello M, Simonelli V, Cardinale A, Merlo D, Dogliotti E (2016) The response to oxidative DNA damage in neurons: mechanisms and disease. Neural Plast 2016: 1–14 10.1155/2016/3619274 PMC4752990 26942017

[embr202050932-bib-0039] Nieuwkoop PD, Faber J (1994) Normal table of *Xenopus laevis* (Daudin) : a systematical and chronological survey of the development from the fertilized egg till the end of metamorphosis.

[embr202050932-bib-0040] Ogai K, Nakatani K, Hisano S, Sugitani K, Koriyama Y, Kato S (2014) Function of Sox2 in ependymal cells of lesioned spinal cords in adult zebrafish. Neurosci Res 88: 84–87 25150399 10.1016/j.neures.2014.07.010

[embr202050932-bib-0041] Qiu X, Hill A, Packer J, Lin D, Ma Y‐A, Trapnell C (2017) Single‐cell mRNA quantification and differential analysis with Census. Nat Methods 14: 309–315 28114287 10.1038/nmeth.4150 PMC5330805

[embr202050932-bib-0042] Rodrigo Albors A, Tazaki A, Rost F, Nowoshilow S, Chara O, Tanaka EM (2015) Planar cell polarity‐mediated induction of neural stem cell expansion during axolotl spinal cord regeneration. Elife 4: e10230 26568310 10.7554/eLife.10230 PMC4755742

[embr202050932-bib-0043] Rost F, Rodrigo Albors A, Mazurov V, Brusch L, Deutsch A, Tanaka EM, Chara O (2016) Accelerated cell divisions drive the outgrowth of the regenerating spinal cord in axolotls. Elife 5: e20357 27885987 10.7554/eLife.20357 PMC5182066

[embr202050932-bib-0044] Rottach A, Kremmer E, Nowak D, Boisguerin P, Volkmer R, Cardoso MC, Leonhardt H, Rothbauer U (2008) Generation and characterization of a rat monoclonal antibody specific for PCNA. Hybridoma 27: 91–98 18642673 10.1089/hyb.2007.0555

[embr202050932-bib-0045] Schüller U, Zhao Q, Godinho SA, Heine VM, Medema RH, Pellman D, Rowitch DH (2007) Forkhead transcription factor FoxM1 regulates mitotic entry and prevents spindle defects in cerebellar granule neuron precursors. Mol Cell Biol 27: 8259–8270 17893320 10.1128/MCB.00707-07 PMC2169184

[embr202050932-bib-0046] Slack JMW, Lin G, Chen Y (2008) The *Xenopus* tadpole: a new model for regeneration research. Cell Mol Life Sci 65: 54–63 18030419 10.1007/s00018-007-7431-1 PMC11131608

[embr202050932-bib-0047] Thuret R, Auger H, Papalopulu N (2015) Analysis of neural progenitors from embryogenesis to juvenile adult in *Xenopus laevis* reveals biphasic neurogenesis and continuous lengthening of the cell cycle. Biol Open 4: 1772–1781 26621828 10.1242/bio.013391 PMC4736028

[embr202050932-bib-0048] Tran HTN, Ang KS, Chevrier M, Zhang X, Lee NYS, Goh M, Chen J (2020) A benchmark of batch‐effect correction methods for single‐cell RNA sequencing data. Genome Biol 21: 12 31948481 10.1186/s13059-019-1850-9 PMC6964114

[embr202050932-bib-0049] Trapnell C, Cacchiarelli D, Grimsby J, Pokharel P, Li S, Morse M, Lennon NJ, Livak KJ, Mikkelsen TS, Rinn JL (2014) The dynamics and regulators of cell fate decisions are revealed by pseudotemporal ordering of single cells. Nat Biotechnol 32: 381–386 24658644 10.1038/nbt.2859 PMC4122333

[embr202050932-bib-0050] Turrero García M, Chang Y, Arai Y, Huttner WB (2016) S‐phase duration is the main target of cell cycle regulation in neural progenitors of developing ferret neocortex. J Comp Neurol 524: 456–470 25963823 10.1002/cne.23801 PMC5008145

[embr202050932-bib-0051] Ueno H, Nakajo N, Watanabe M, Isoda M, Sagata N (2008) FoxM1‐driven cell division is required for neuronal differentiation in early *Xenopus* embryos. Development 135: 2023–2030 18469223 10.1242/dev.019893

[embr202050932-bib-0052] Wu X, Gu X, Han X, Du A, Jiang Y, Zhang X, Wang Y, Cao G, Zhao C (2014) A novel function for Foxm1 in interkinetic nuclear migration in the developing telencephalon and anxiety‐related behavior. J Neurosci 34: 1510–1522 24453338 10.1523/JNEUROSCI.2549-13.2014 PMC6705310

[embr202050932-bib-0053] Wu YE, Pan L, Zuo Y, Li X, Hong W (2017) Detecting activated cell populations using single‐cell RNA‐Seq. Neuron 96: 313–329 29024657 10.1016/j.neuron.2017.09.026

