# Supplementary material for: Foxm1 regulates neural progenitor fate during spinal cord regeneration
Source: EMBO Rep. 2021 Aug 24;22(9):e50932. doi: 10.15252/embr.202050932 (PMC8419688; doi:10.15252/embr.202050932)
Supplement: Supplementary file 2 — Appendix [file EMBR-22-e50932-s003.pdf]

Appendix for "Foxm1 regulates neural progenitor fate during spinal cord regeneration"

Table of contents of Appendix figures

Appendix Figure S1	page 1
Appendix Figure S2	page 2
Appendix Figure S3	page 3
Appendix Figure legends	page 4 and 5

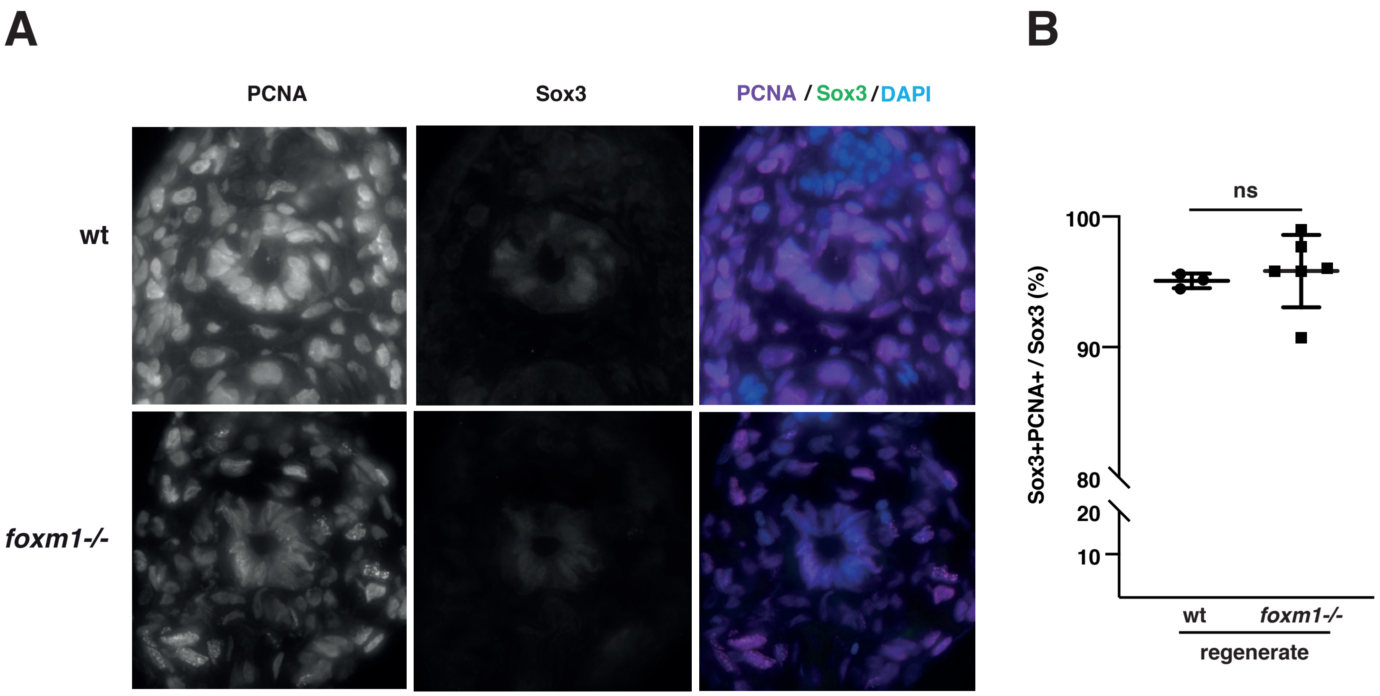

Appendix Figure S1 Pelzer *et al.*

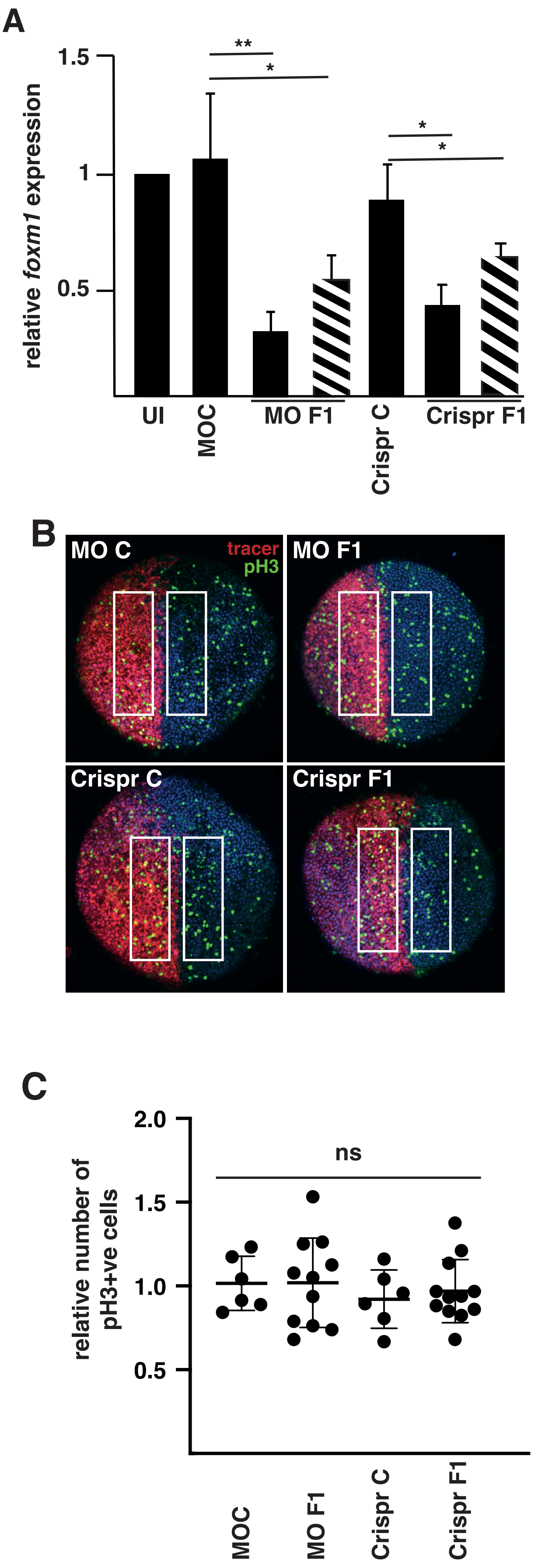

Appendix Figure S2 Pelzer *et al*.

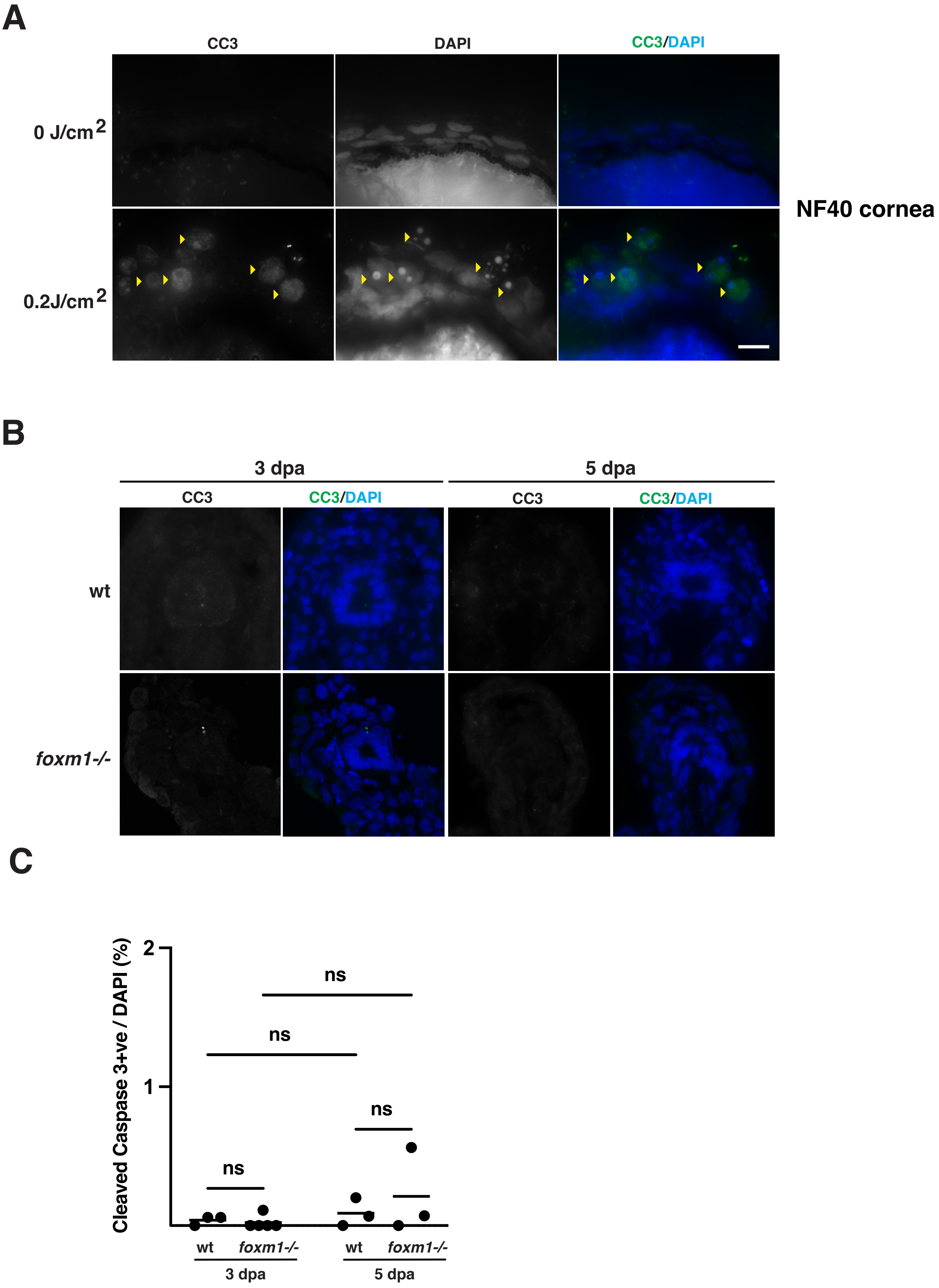

Appendix Figure S3 Pelzer *et al.*

Appendix Figure S1: estimation of the growth fraction at 3 dpa

(A) NF50 spinal cord sections at 3 dpa from wt and *foxm1-/-* tadpoles were immunostained with antibodies against PCNA and Sox3.

(B) Quantification of images as in (A) The graph represents the mean +/- SD of 3 to 6 tadpoles with an average of 7 sections per tadpole.

Appendix Figure S2: Effect of *foxm1* knock-down during primary neurogenesis

(A) Embryos were injected with morpholino specific for *foxm1* (MO F1), morpholino control (MO C), Cas9 with gRNA against *foxm1* (Crispr F1) or Cas 9 alone (Crispr C) at the 1-cell stage (black bars) or in one cell of a 2-cell stage embryo (dashed bars). Embryos were collected at NF13, total RNA isolated and the level of *foxm1* expression analysed by qPCR using *ef1*α as a reference.

(B) Embryos were injected at the 2-cell stage with the indicated compounds as in (A) together with Dextran-Rhodamine as a tracer (red). At NF13, embryos were fixed in MEMFA and process for immunostaining using anti-phospho-Histone 3 (pH3) antibodies (green). The images are maximal z-projections of confocal sections and the white rectangle show the area used for pH3 quantification.

(C) Quantification of the images presented in (B).

Data information: The graphs represent the mean +/- SD analysed using a one-way ANOVA. ns: non-significant, *p<0.05, ** p<0.01

Appendix Figure S3 Quantification of apoptosis during spinal cord regeneration

(A) Validation of anti - Cleaved-Caspase 3 antibodies. NF40 tadpoles were exposed (0.2 J/cm2) or not (0 J/cm2) to UVC light. Embryos were fixed at 2.5 hours post-UV exposure

and processed for staining with anti-Cleaved Caspase 3 (CC3, active Caspase 3) antibodies (green) and DAPI staining (blue). The scale bar is 10*μ*m.

(B) Staining of the regenerating spinal cord with anti-CC3 antibodies. The tails of wildtype (wt) and *foxm1* knockout (*foxm1-/-)* of NF50 tadpoles were amputated. The tails were fixed at 3 and 5 dpa and processed for anti-CC3 (green) and DAPI (blue) staining).

(C) Quantification of the images in (B). The graph represents the mean of 3 independent experiments with an average of 8 sections per tadpole.
