# Supplementary material for: Foxm1 regulates neural progenitor fate during spinal cord regeneration
Source: EMBO Rep. 2021 Aug 24;22(9):e50932. doi: 10.15252/embr.202050932 (PMC8419688; doi:10.15252/embr.202050932)
Supplement: Supplementary file 3 — Expanded View Figures PDF [file EMBR-22-e50932-s004.pdf]

Expanded View Figures

Figure EV1. Metadata from the RNA-seq experiment of the time course of isolated spinal cord regeneration.

A Principal component analysis to assess overall similarities between all samples. The biological replicates of day 0 (0 dpa, green square), day 1 (1 dpa, red circle) and day 3 post-amputation (3 dpa, blue triangle) cluster together whilst showing wide variation in the two dimensions shown on the graph.

B Hierarchical clustering of the nine datasets.

C, D MA plots depicting the log2 fold change against the mean of normalised counts. DE genes (P_adj< 0.05) are coloured in red when comparing day 0 versus day 1 (C) and day 0 versus day 3 (D).

E Total number of differentially up- and downregulated (ILog2(FC)I> 1, P_adj< 0.01) transcripts in 0 dpa versus 1 dpa and 0 dpa versus 3 dpa samples.

F Schematic of the experiment designed to identify the signals upstream of *foxm1* expression. After amputation, the tails were left to heal for 36h before inhibitor treatments were started. The tails were collected at 72hpa, and *foxm1* expression was determined by RT–qPCR.

G–I Effects of treating tadpoles with 4 μM DPI (a NOX inhibitor, G), 20 μM SU5402 (an FGFR inhibitor, H) and 2.5 μM cyclopamine (a Hedgehog signalling inhibitor, I) on *foxm1* expression. DMSO was used as a control for G and H and ethanol for I.

Data presentation: The graphs in G–I represent the mean with standard deviation of four independent experiments with at least 15 tails per experiments, *ef1α* was used to normalise expression, and significance was assessed with an unpaired t-test, *$P < 0.05$.

© 2021 The Authors

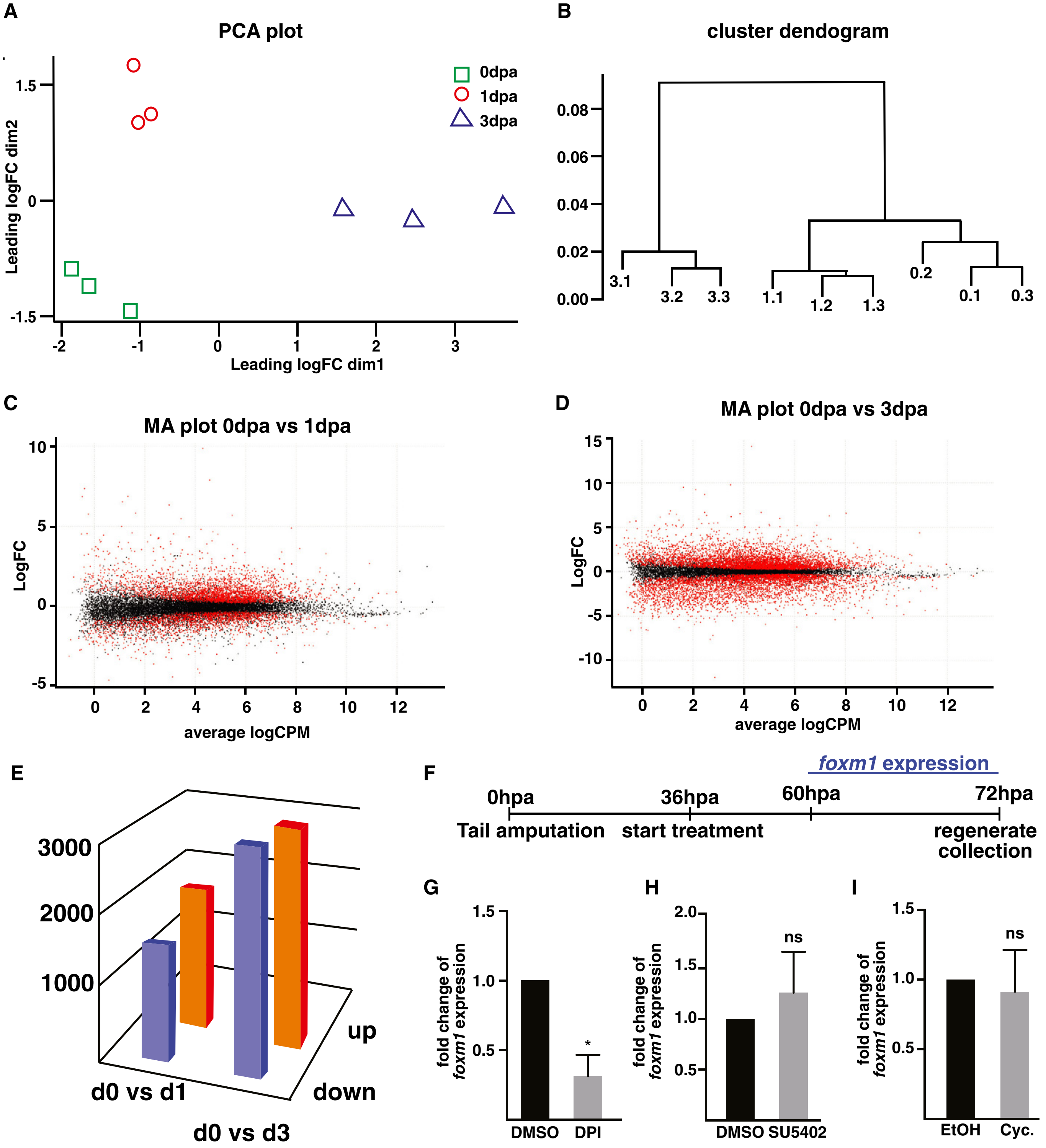

Figure EV1.

 © 2021 The Authors

Figure EV2. Establishment of a *foxm1* knockout line.

A The CRISPR/Cas9 system was used to generate *foxm1* knockdown and knockout animals, and gRNA was designed to target the *foxm1* gene. The target region contains the restriction site for NcoI and was used to test efficiency by RFLP.

B Embryos were either uninjected (UI) or coinjected with gRNA and Cas9mRNA, 0.6 ng Cas9 protein or 1.5 ng Cas9 protein. Genomic DNA was extracted and a region amplified around the gRNA target site by PCR. Half of the PCR product was digested with NcoI. By comparing the ratio of the digested product with an intact restriction site (lower band) to the non-digested product containing a mutated restriction site (upper band) after the addition of NcoI (+) gives an indication of the efficiency of the induction of mutations.

C Frogs injected with the CRISPR/Cas9 system and raised to adulthood. The F1 embryos were sequenced for mutations in *foxm1*. Four frameshift mutations were identified.

D Genotypes used in this study.

E Tadpoles from a *foxm1*$^{+/-}$ cross were raised to NF50, amputated and the tails collected at 3dpa for RNA expression and the heads for genotyping. *Foxm1* expression was analysed by qPCR, using *ef1α* as a reference ($n = 3$ with at least 3 embryos per sample). The data are expressed as the mean ± SD.

F A third of the tails of *foxm1* knockdown (Crispr mosaic F0) and wt tadpoles at NF50 were amputated and the tadpoles left to regenerate for 9 days. The images show representative tails at 9dpa.

G, H To quantify the rate of regeneration, the ratio of the length of the regenerate to the length that has originally be amputated was compared for the spinal cord (G) and the whole tail (H). The graph represents the mean ± SD of three independent experiments with at least five tadpoles in each experiment.

Data presentation: For testing statistical significance, an unpaired *t*-test was used in E and a two-way ANOVA followed by a Sidak multiple comparison test in G and H. *$P < 0.05$ and ****$P < 0.0001$.

© 2021 The Authors

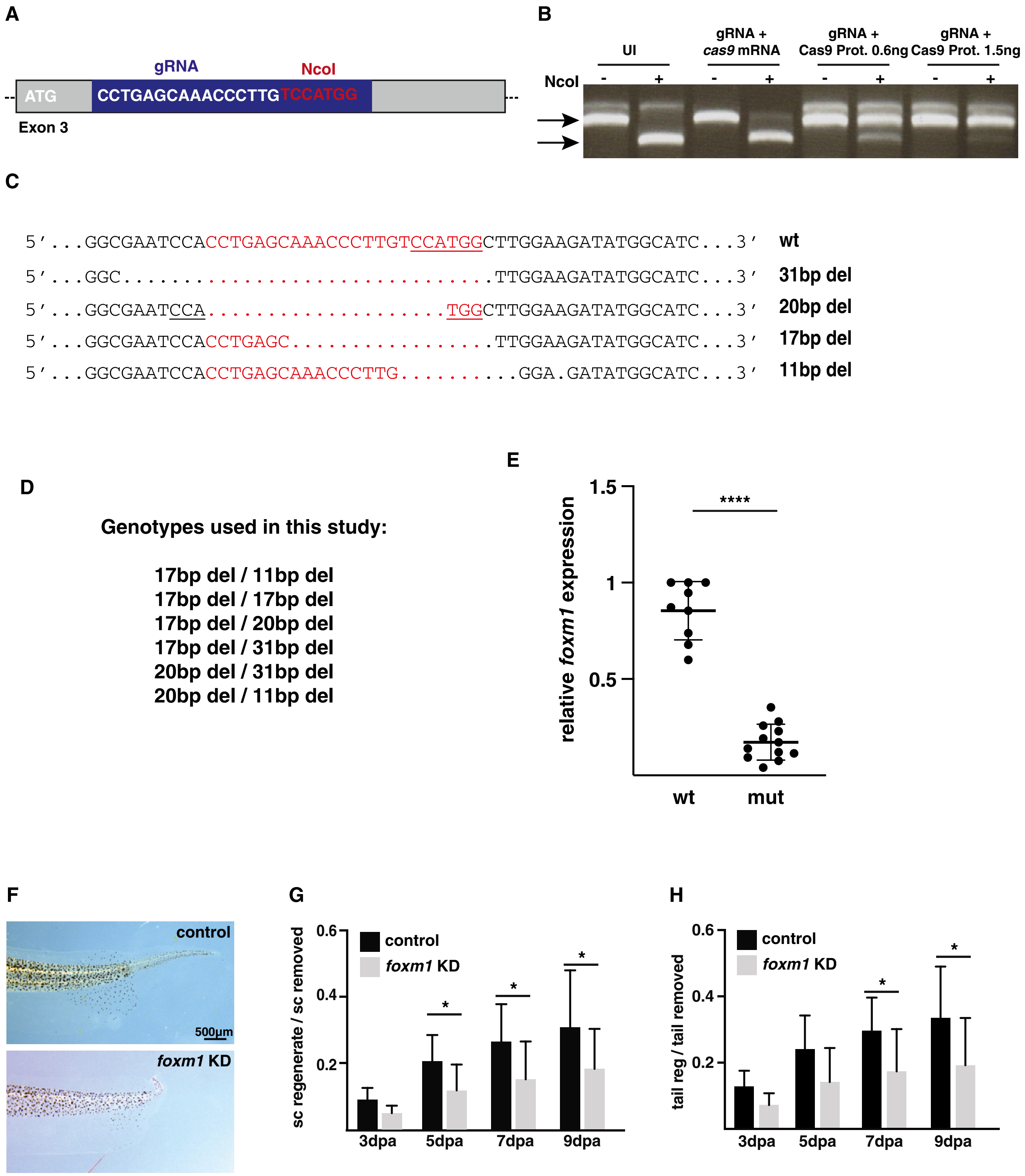

Figure EV2.

 © 2021 The Authors

A

Sample ID	0dpa	3dpa
Stage	50	50
Days post amputation	0	3
Sequencing Batch	1	1
Number of cells	2908	2503
Mean reads per cell	75,815	87,857
Median genes per cell	590	1548
Median UMI counts per cell	2,679	7,109
Total genes detected	16,839	18,870
Fraction reads in cells (%)	88	88.5

B

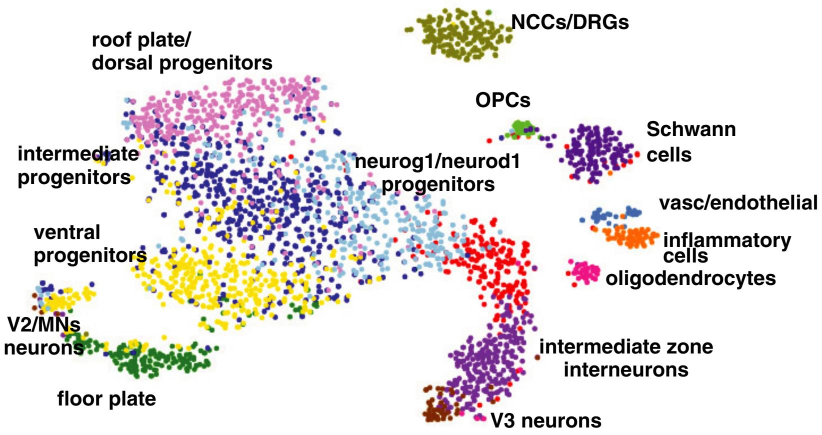

C

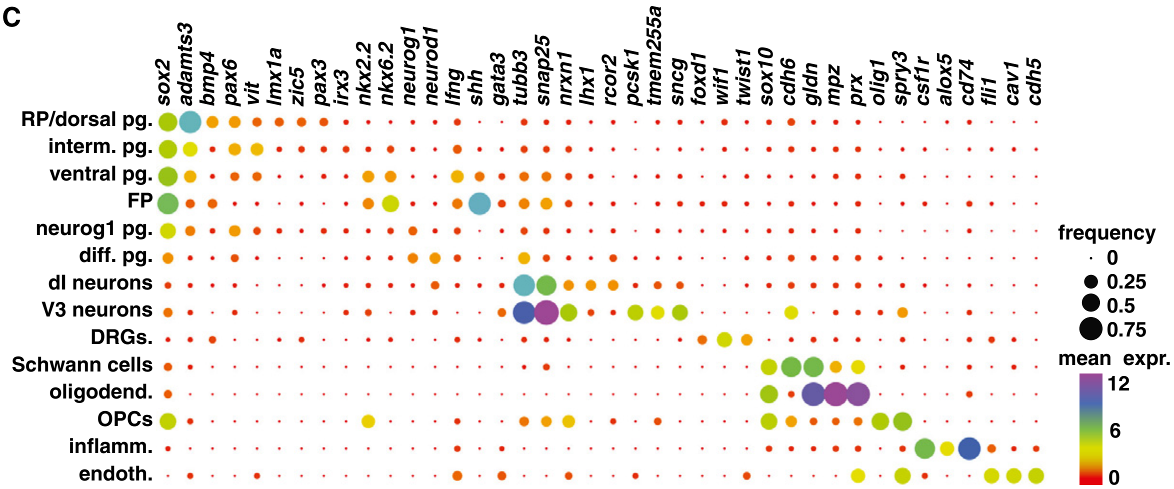

Figure EV3. Characterisation of the *Xenopus* spinal cord by single-cell RNA sequencing.

A Metadata of the scRNA-seq experiment.

B t-SNE representation of the dataset from 0 dpa with the different cell types identified using a dynamic tree cut algorithm.

C Bubble plot representing the proportion of cells (size of the dot) and level of expression (colour of the dot) for the genes used to identify the cell types in (B).

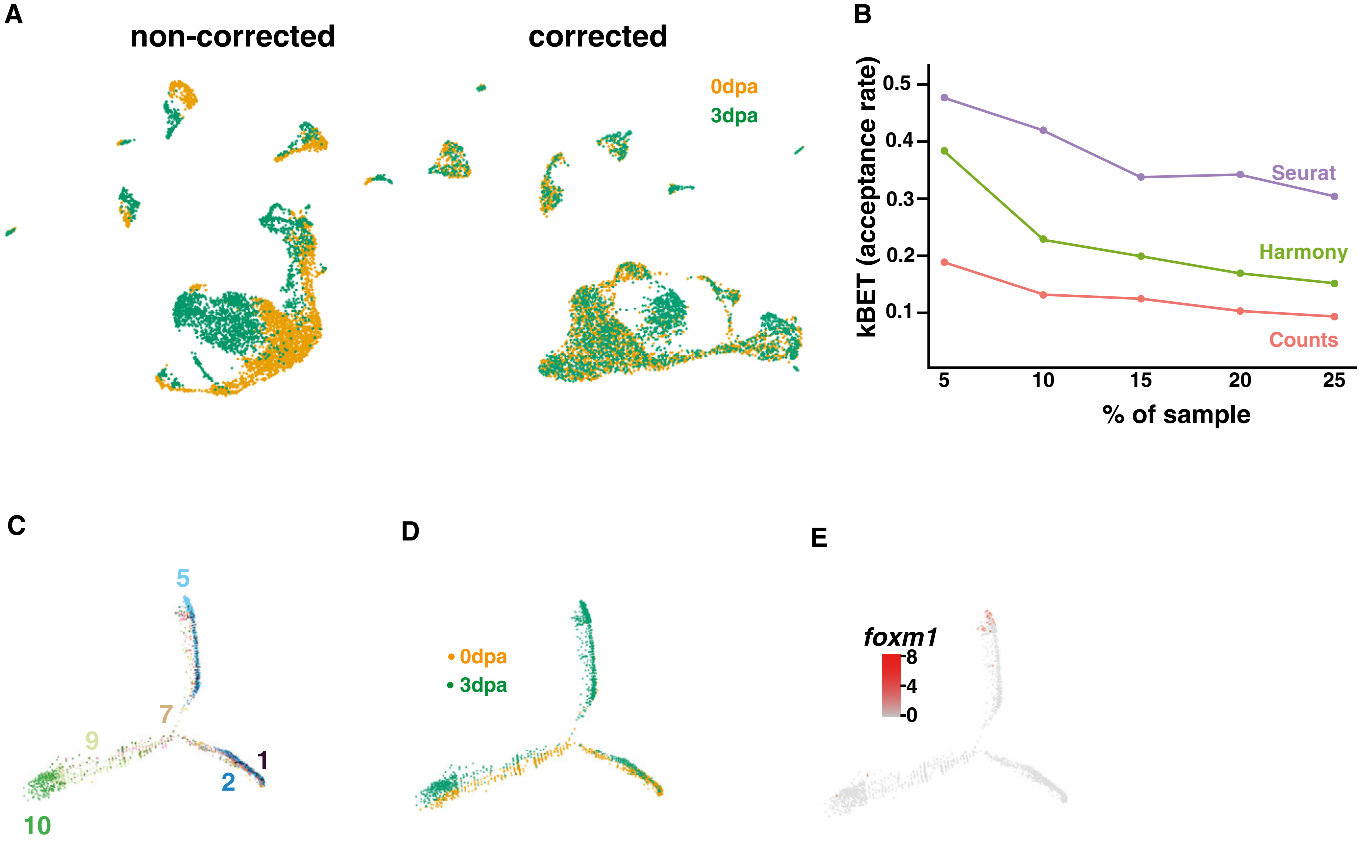

Figure EV4. Characterisation of the *foxm1* positive cells.

A UMAP representation of the scRNA-seq dataset before (left panel) and after (right panel) batch correction using Seurat.

B Unbiased acceptance rate at the indicated subsampling percentile in the raw data (Counts) and after batch correction using Harmony or Seurat algorithm.

C Unsupervised pseudo-time of the whole scRNA-seq dataset. The distribution of the different clusters along the pseudo-time is indicated with the colours and numbers as described in Fig 3F.

D Pseudo-time of the whole scRNA-seq dataset with cells from 0 dpa in orange and from 3 dpa in green.

E Pseudo-time representation showing the cells expressing *foxm1* (red dots).

 © 2021 The Authors

Figure EV5. Effect of impairing *foxm1* expression on the organisation of the regenerating spinal cord.

A Tails from tadpoles *foxm1* knockdown (mosaic Crispr F0, kd) and control (wt) NF50 tadpoles were amputated and left to regrow for 3 days. RNA was isolated from the regenerates and expression levels of *sox2* and *ntubulin* analysed by qPCR using *ef1*α as a reference. *sox2*: $n = 4$, *ntubulin*: $n = 6$, with at least 20 tails per sample,

B Effect of DPI treatment on the expression of known transcriptional targets of Foxm. Embryos were treated with DPI as described in Fig EV1F, and the expression of *ccnb3* (a Foxm1 target gene), *ntub* (a marker of differentiated neurons) and *ami* (a gene expressed in endothelial cells) was analysed by RT–qPCR using *ef1α* as control.

C, D Rose plot histograms showing the percentage frequency distribution of the angles of $DAPI^+$ nuclei (C) or $Sox3^+$ nuclei (D) in wt (blue) or *foxm1*$^{-/-}$ spinal cords (red). Angles are distributed into 12 bins from 0 to 180 degrees using a MATLAB script. Dorsal = 0 degrees, lateral = 90 degrees and ventral = 180 degrees. The inner, middle and outer circle corresponds to 5, 10 and 15%, respectively. Ten sections from $n = 4$ animals were analysed per genotype. Total cell counts were as follows: wt anterior ($DAPI^+$ 2166; $Sox3^+$, 701), *foxm1*$^{-/-}$ anterior ($DAPI^+$, 2246; $Sox3^+$, 707), wt regenerate ($DAPI^+$, 1927 nuclei; $Sox3^+$, 826) and *foxm1*$^{-/-}$ regenerate ($DAPI^+$, 2393; $Sox3^+$, 1125). $P < 0.0001$ for regenerate spinal cords and $P > 0.05$ for anterior spinal cords as analysed by Kolmogorov–Smirnov tests.

E, F Quantification of the absolute number of cells per section expressing Sox3 (E) and nuclei (DAPI, F) in the regenerate of wild type (wt) and *foxm1*$^{-/-}$ knockout tadpoles at 5 dpa. The number is derived from the same sections analysed in Fig 5C, and the quantification is derived from the analysis of 8 tadpoles with an average of 15 sections per tail.

G The tails of control and *foxm1*KD animals, fixed at 5 days post-amputation, were sectioned and labelled with the Sox3 or Myt1 antibody followed by DAPI staining. The ratio of Sox3 and Myt1 per number of DAPI stained nuclei in the spinal cord was quantified and compared between control and foxm1KD tadpoles. Sox3 wt $n = 6$ with 41 sections, CRISPR/Cas9 $n = 5$ with 50 sections, Myt1 wt = 8 with 67 sections and foxm1KD $n = 7$ with 53 sections.

Data information: In A and B, the graph represents the mean ± SD of three independent experiments normalised to wt. In E and F, the central line represents the median, the box the $25^{th}/75^{th}$ percentile and the whiskers the min and max values. In G, the graph shows the mean ± SD. For A, E, F and G, the significance was tested with an unpaired *t*-test, and for B, a one-way ANOVA with a Tukey post hoc test was used. ns: non-significant, *$P < 0.05$, ***$P < 0.001$ and ****$P < 0.0001$.

© 2021 The Authors

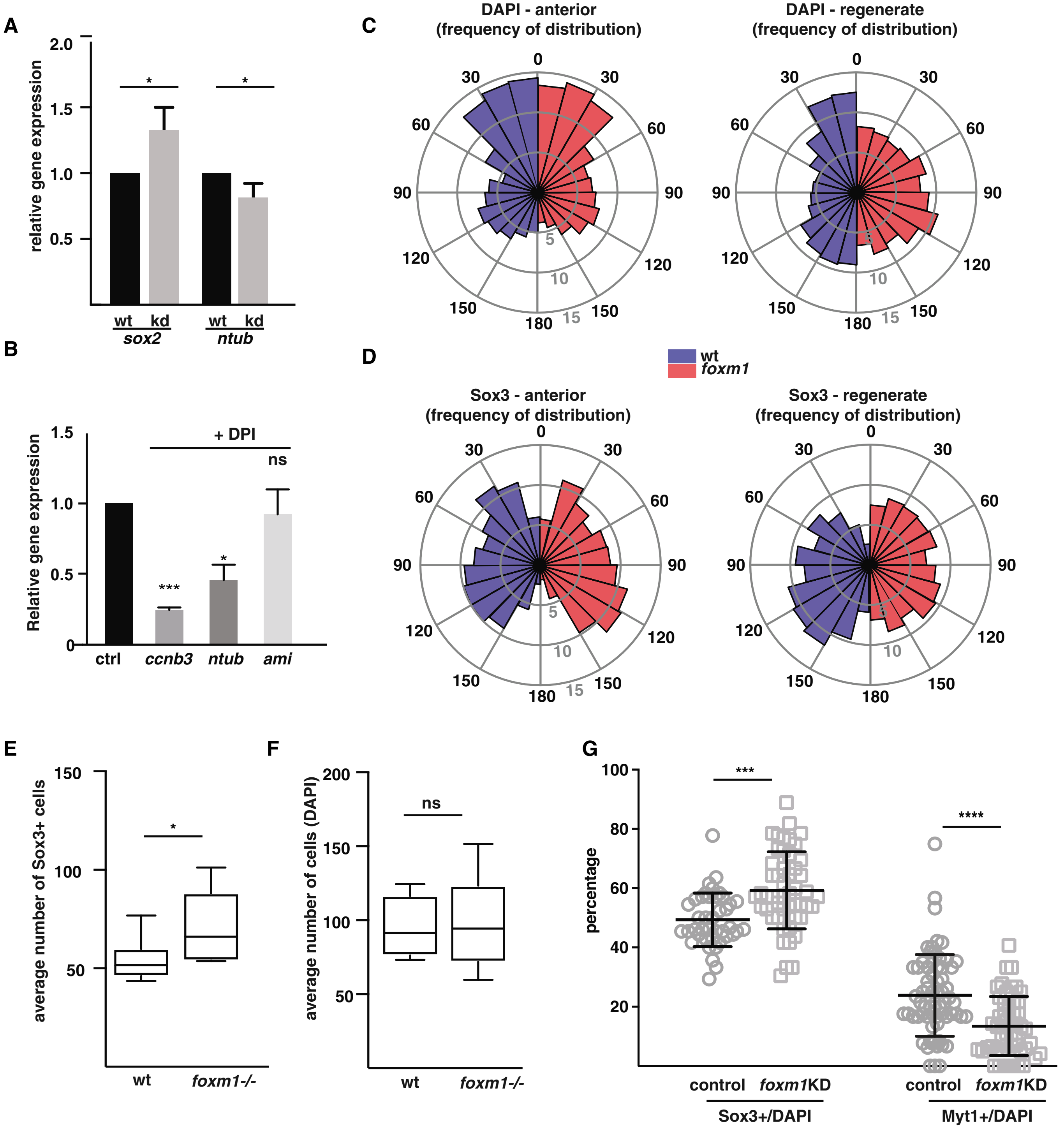

Figure EV5.

© 2021 The Authors
